# Supplementary material for: Lutein derived from Xenostegia tridentata exhibits anticancer activities against A549 lung cancer cells via hyaluronidase inhibition
Source: PLoS One. 2024 Dec 16;19(12):e0315570. doi: 10.1371/journal.pone.0315570 (PMC11649105; doi:10.1371/journal.pone.0315570)
Supplement: S1 Table — (PDF) [file pone.0315570.s001.pdf]

S1. Crystallographic data of compound 1

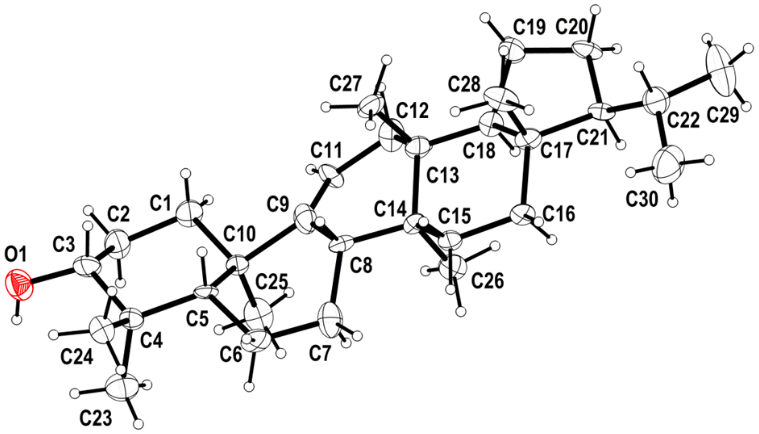

Figure 1.. Asymmetric unit of Compound **1**

Table S1. Crystal data and structure refinement for compound 1.

Identification code	**Compound 1**
CCDC No.	2334660
Empirical formula	$C_{30}H_{50}O$
Formula weight	426.70
Temperature/K	293(2)
Crystal system	monoclinic
Space group	*C*2
a/Å	36.695(4)
b/Å	7.6196(15)
c/Å	10.733(2)
β/°	92.017(15)
Volume/Å^3	2999.1(9)
Z	4
Crystal size/mm^3	0.2 × 0.05 × 0.05
Radiation	MoKα (λ = 0.71073)
2*θ* range for data collection/°	3.798 to 54.462
Index ranges	$-38 \le h \le 45$, $-4 \le k \le 4$, $-13 \le l \le 12$
Reflections collected	3706
Independent reflections	2628 [R_{int} = 0.0752, R_{sigma} = 0.1630]
Data/restraints/parameters	2628/227/290
Goodness–of–fit on F^2	0.858
Final *R* indexes [I ≥ 2σ (I)]	R_1 = 0.0897, wR_2 = 0.2260
Final *R* indexes [all data]	R_1 = 0.1879, wR_2 = 0.3009
Largest diff. peak/hole / e Å^{-3}	0.16/–0.20
Flack parameter	–3.8(10)

[a] $R_1 = \sum||F_o| - |F_c||/\sum|F_o|$ and $wR_2 = [\sum w(F_o^2 - F_c^2)^2/\sum wF_o^4]^{1/2}$ for $F_o^2 > 2\sigma(F_o^2)$.
