# Supplementary material for: Lutein derived from Xenostegia tridentata exhibits anticancer activities against A549 lung cancer cells via hyaluronidase inhibition
Source: PLoS One. 2024 Dec 16;19(12):e0315570. doi: 10.1371/journal.pone.0315570 (PMC11649105; doi:10.1371/journal.pone.0315570)
Supplement: S1 Fig — (PDF) [file pone.0315570.s002.pdf]

S2. Spectroscopic data of the isolated compounds

1. Compound 1 (Fernenol)

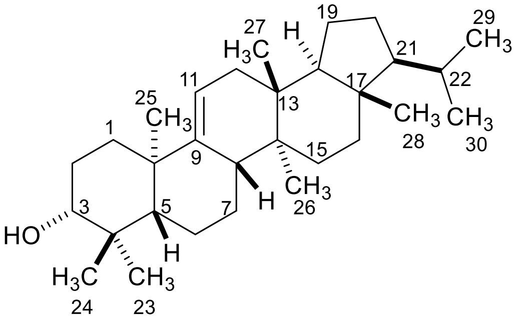

position	DOI 10.1016/S0040-4020(01)86999-4		Compound 1	
	^{13}C-NMR (125 MHz)	^{1}H-NMR (500 MHz)	^{13}C-NMR (75 MHz)	^{1}H-NMR (300 MHz)
1	39.35		39.49	
2	28.14	1.58, 1.64	28.28	
3	79.16	3.210 (dd, J=10.6, 5.5 Hz)	79.29	3.21 (t, J = 8.0 Hz)
4	39.28		39.41	
5	44.3		44.43	
6	19.15		19.30	
7	18	1.34, 1.59	18.10	
8	39.99		40.13	
9	151.07		151.20	
10	37.66		37.80	
11	116.2	5.296 (ddd, J=5.2, 2.5, 2.5 Hz)	116.33	5.30 (dt, J=5.1, 2.5 Hz)
12	36.74		36.87	
13	36.74		36.87	
14	37.79		37.93	
15	29.28		29.43	
16	36.16		36.30	
17	42.95		43.08	
18	51.97		52.11	
19	20.14		20.28	
20	28.21	1.23, 1.84	28.36	1.83 (m)
21	59.66		59.80	
22	30.78		30.92	
23	27.45	0.963	27.60	0.96
24	15.05	0.87	15.19	0.87
25	25.22	1.065	25.36	1.06
26	15.85	0.732	16.00	0.73
27	15.38	0.813	15.52	0.81
28	13.99	0.757	14.13	0.76
29	22.13	0.891 (d, J=6.4 Hz)	22.27	0.89 (d, J=6.4 Hz)
30	23	0.830 (d, J=6.4 Hz)	23.15	0.85 (d, J=6.4 Hz)

Compound 1: ^{1}H NMR (300 MHz, $CDCl_3$) δ 5.30 (dt, *J* = 5.1, 2.5 Hz, 1H, H-11), 3.21 (t, *J* = 8.0 Hz, 1H, H-3), 2.12 – 1.97 (m, 1H), 1.93 (dt, *J* = 13.4, 3.5 Hz, 1H), 1.87-1.79 (m, 1H, H-20), 1.78 – 1.27 (m, 18H), 1.25 (s, 2H), 1.06 (s, 3H, H-25), 1.00 (m, 1H), 0.96 (s, 3H, H-23), 0.89 (d, *J* = 6.4 Hz, 3H, H-29), 0.87 (s, 3H, H-24), 0.85 (d, *J* = 6.4 Hz, 3H, H-30), 0.81 (s, 3H, H-27), 0.76 (s, 3H, H-28), 0.73 (s, 3H, H-26). ^{13}C NMR (75 MHz, $CDCl_3$) δ 151.20, 116.33, 79.29, 59.80, 52.11, 44.43, 43.08, 40.13, 39.49, 39.41, 37.93, 37.80, 36.87, 36.87, 36.30, 30.92, 29.43, 28.36, 28.28, 27.60, 25.36, 23.15, 22.27, 20.28, 19.30, 18.10, 16.00, 15.52, 15.19, 14.13. $C_{30}H_{51}O$: $[M+H]^+$ calc: 427.39344; found: 427.3933 m/z

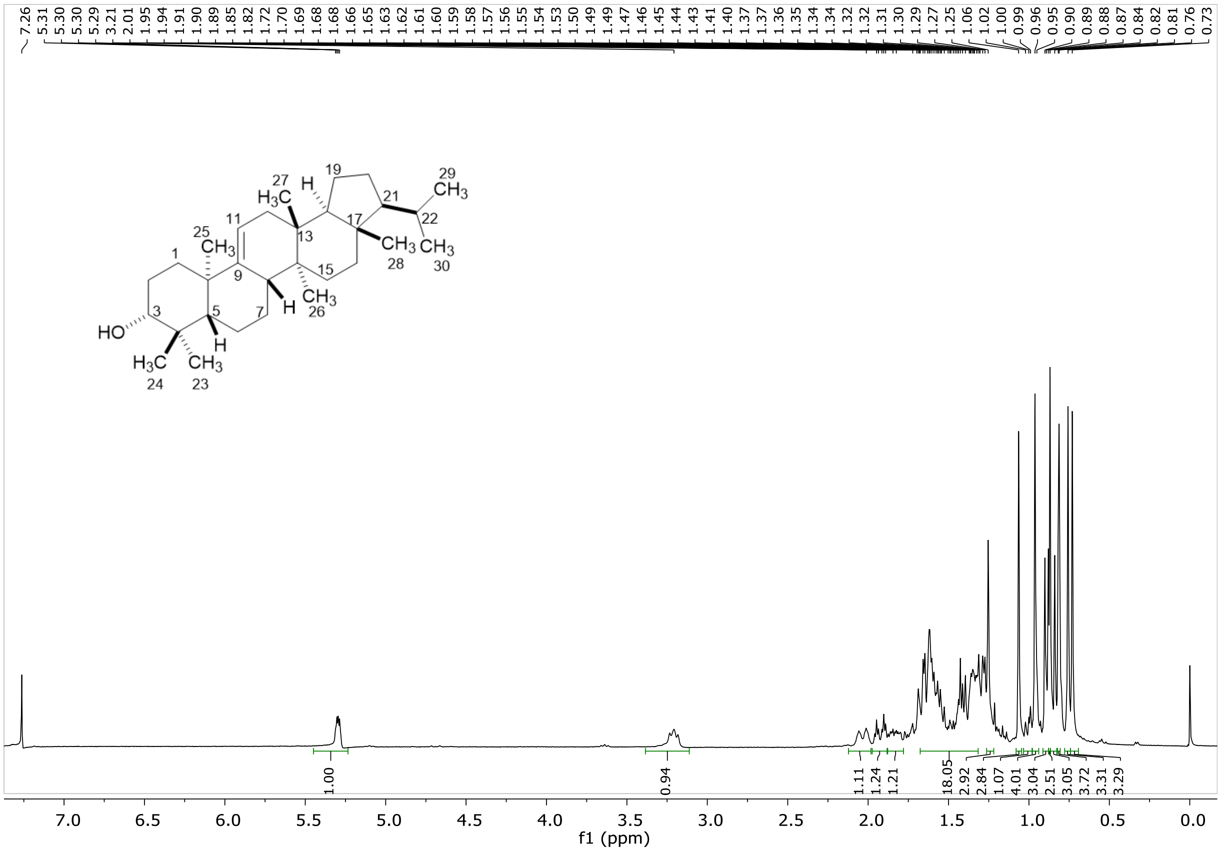

Figure S2.1: ^{1}H NMR spectrum (300 MHz, Chloroform-*d*) of compound 1 (Fernenol)

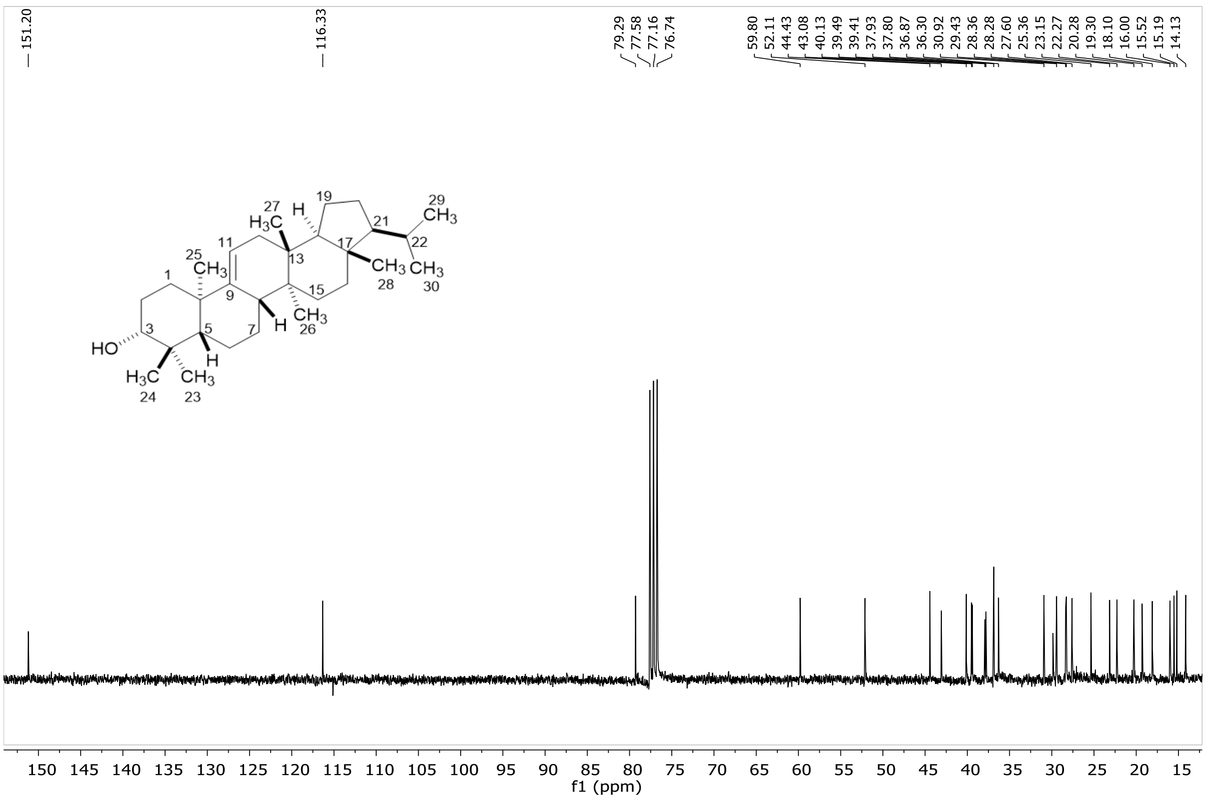

Figure S2.2: ^{13}C NMR spectrum (75 MHz, Chloroform-*d*) of compound 1 (Fernenol)

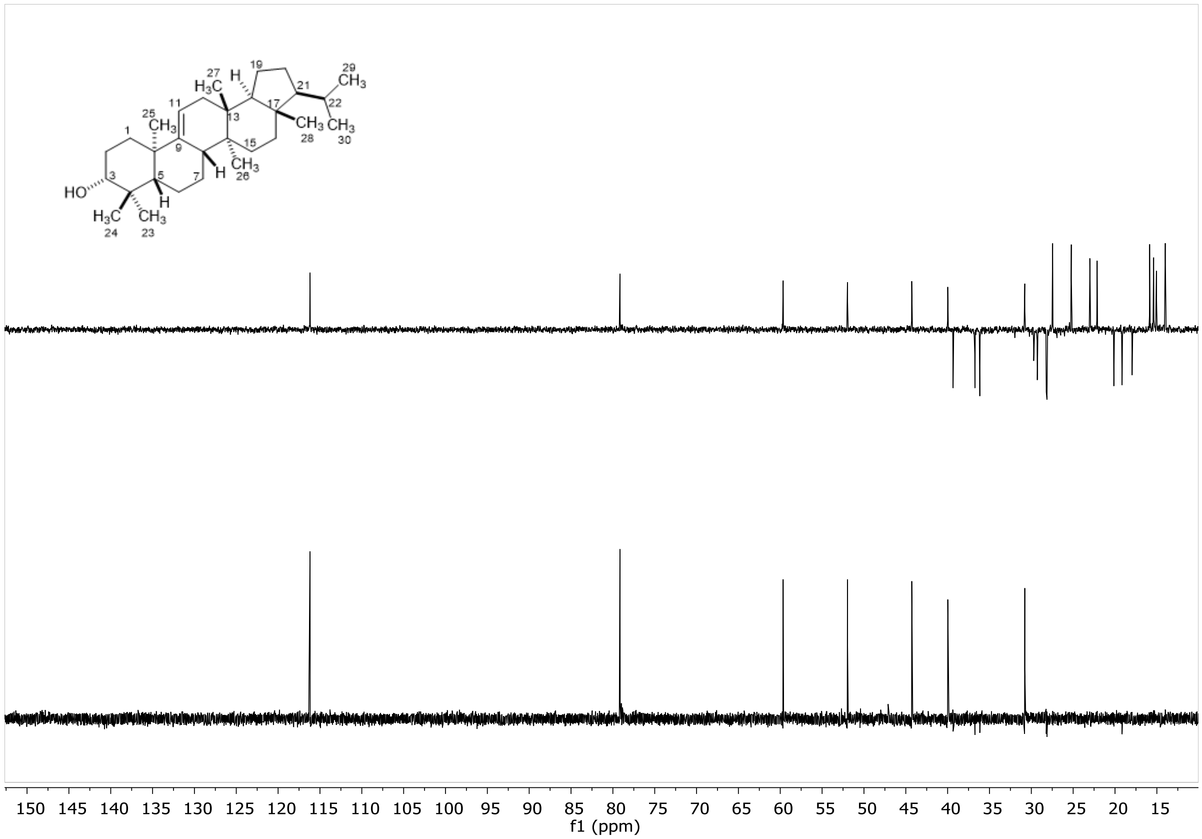

Figure S2.3: DEPT135 and DEPT90 spectra of compound 1 (Fernenol)

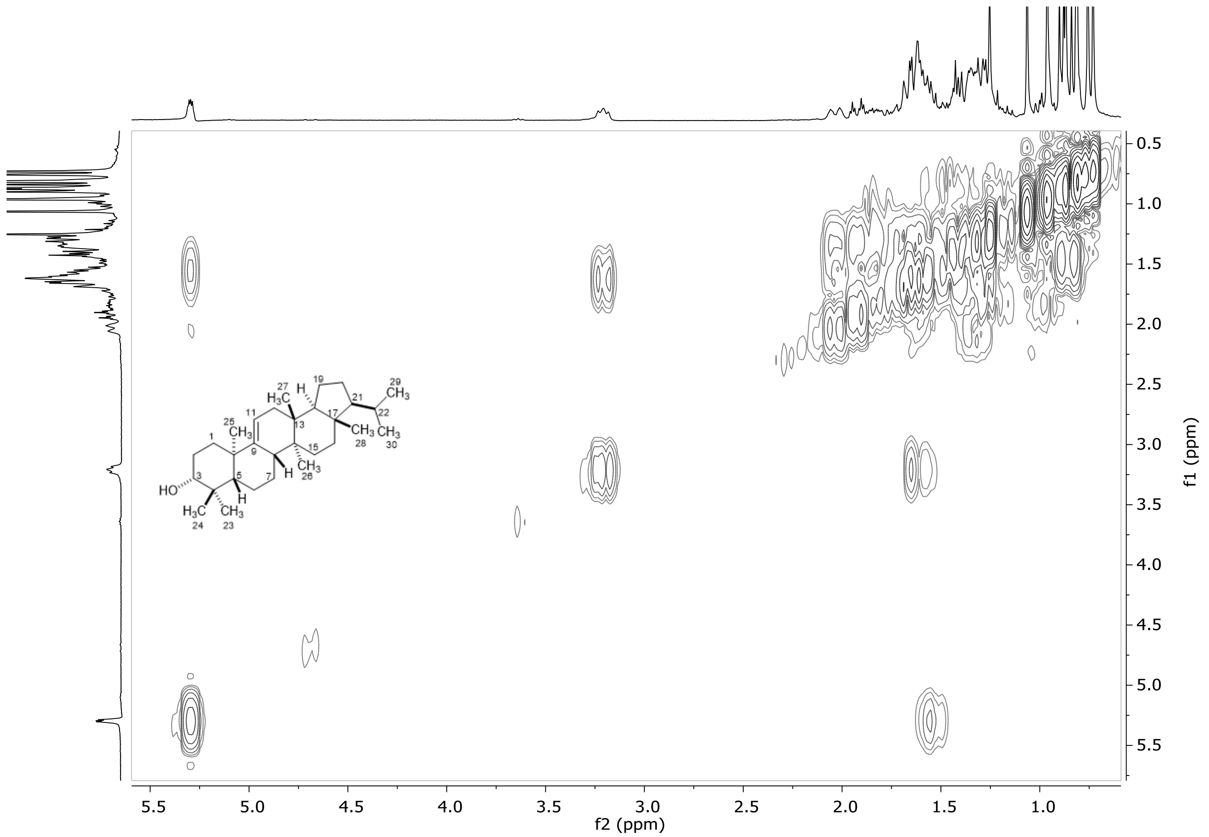

Figure S2.4: COSY spectrum of compound 1 (Fernenol)

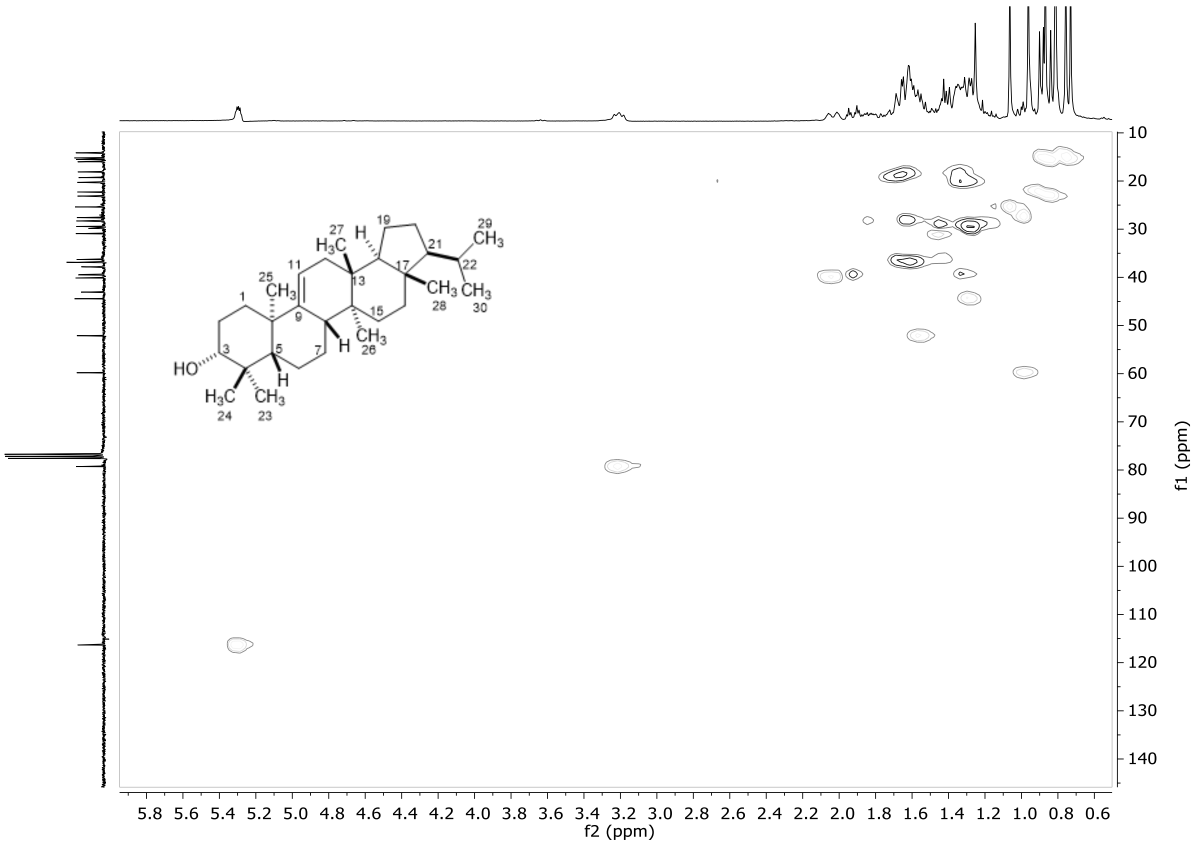

Figure S2.5: HSQC spectrum of compound 1 (Fernenol)

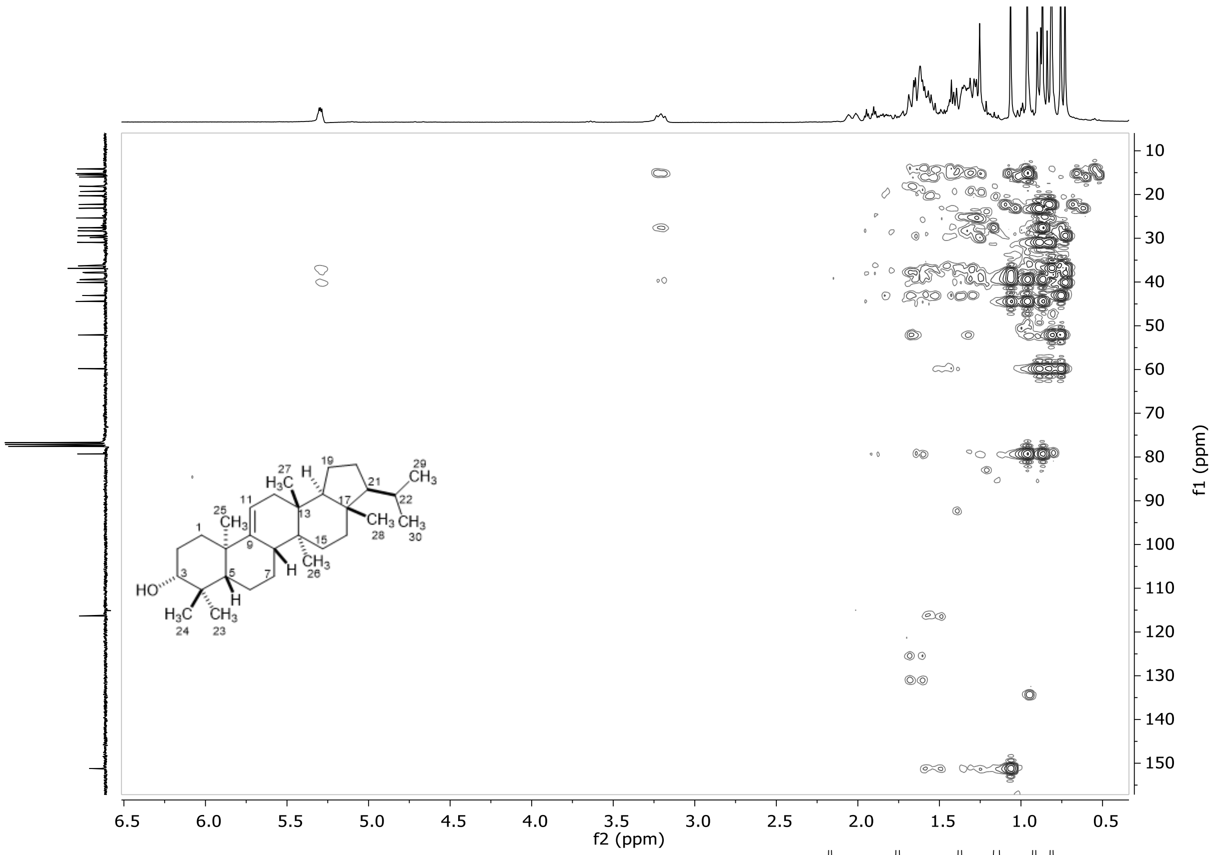

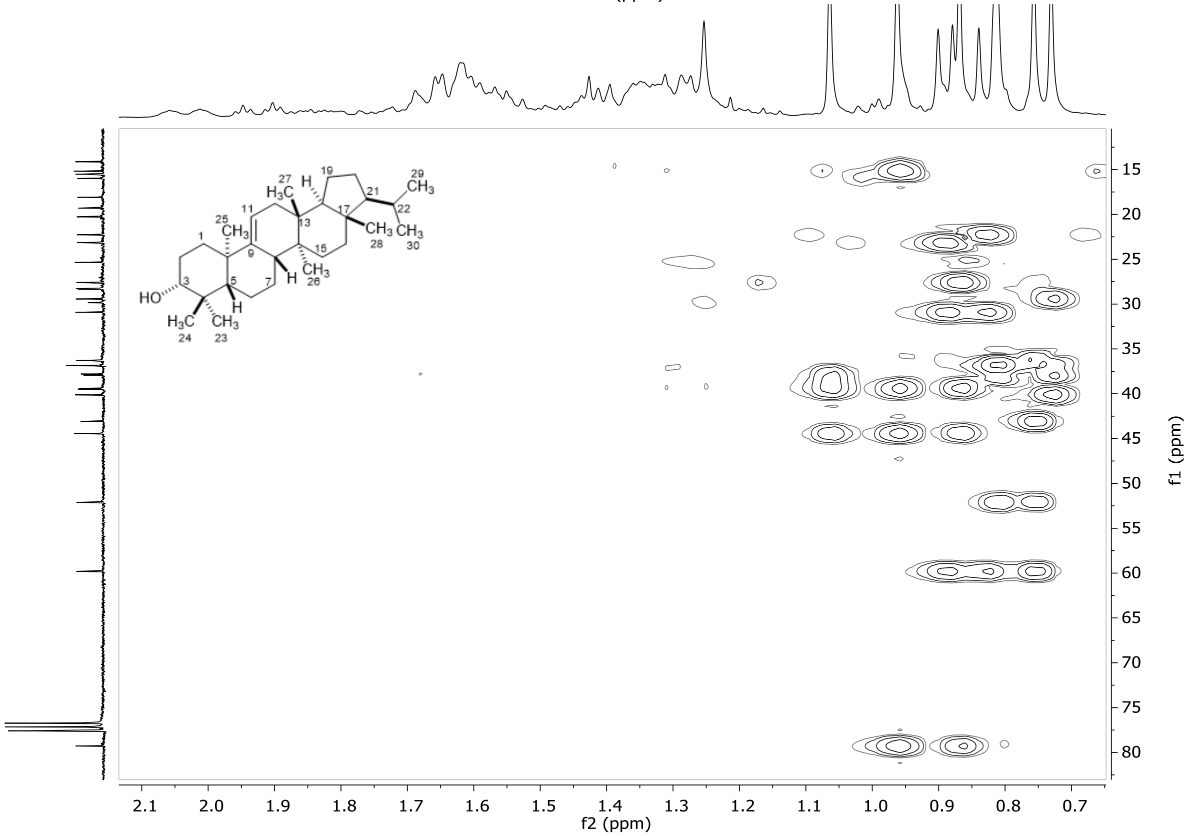

Figure S2.6: HMBC spectrum of compound 1 (Fernenol)

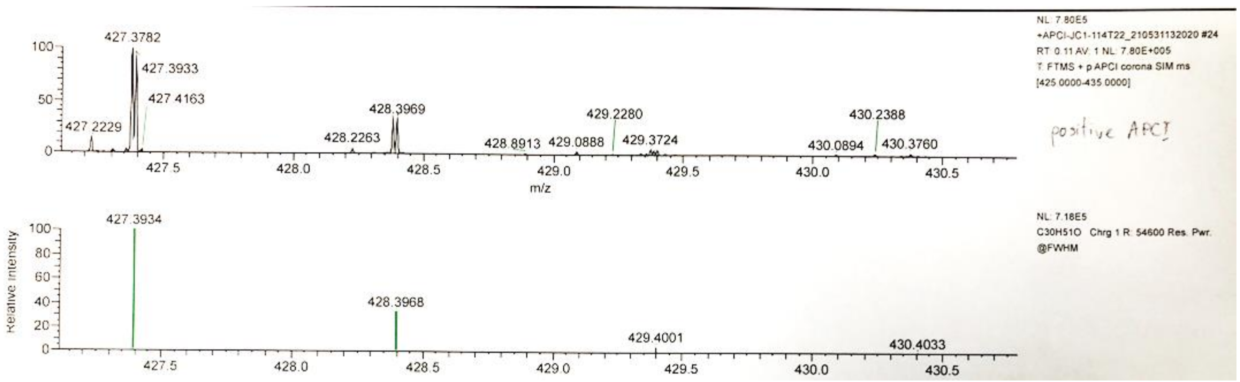

Figure S2.7: HRMS spectrum of compound 1 (Fernenol)

2. Compound 2 (Methyl-3,4-seco-8βH-fernadienoate)

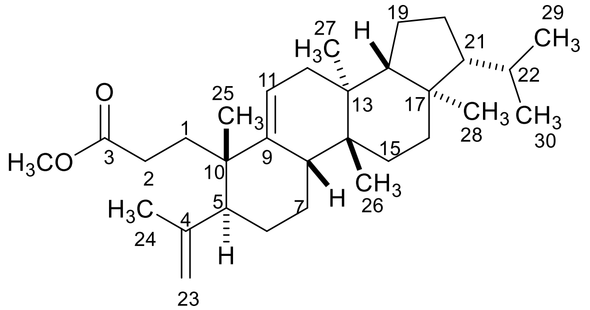

Proton	Reference: DOI 10.1016/0031-9422(95)00721-0	Compound 2
	^{1}H-NMR (400 MHz, $CDCl_3$) δ, ppm	^{1}H-NMR (300 MHz, $CDCl_3$) δ, ppm
H-11	5.32 (ddd, J=5.8, 2.0, 2.0 Hz)	5.32 (d, J = 5.7 Hz, 1H)
H-23	4.78 (d, J=1.8 Hz)	4.78 (s, 1H)
	4.89 (t, J= 1.8 Hz)	4.89 (d, J = 2.0 Hz, 1H)
H-24	1.71	1.71 (s, 3H)
H-25	1.01	1.01 (s, 3H)
H-26	0.77	0.77 (s, 3H)
H-27	0.84	0.84 (s, 3H)
H-28	0.75	0.75 (s, 3H)
H-29	0.89 (d, J=6.5 Hz)	0.89 (d, J = 6.5 Hz, 3H)
H-30	0.83 (d, J=6.5 Hz)	0.89 (d, J = 6.5 Hz, 3H)
CO2Me	3.65	3.65 (s, 3H)

carbon	Reference: DOI 10.1016/0031-9422(95)00721-0	Compound 2	Δδ (ppm)
	^{13}C-NMR (125 MHz, $CDCl_3$) δ, ppm	^{13}C-NMR (75 MHz, $CDCl_3$) δ, ppm	
1	36.4	36.08	-0.32
2	29.6	29.64	0.04
3	175.5	175.13	-0.37
4	146.5	146.25	-0.25
5	51.1	51.11	0.01
6	25.7	24.27	-1.43
7	19.4	19.52	0.12
8	40.5	40.51	0.01
9	142.3	142.03	-0.27
10	41.3	41.36	0.06
11	119.4	119.22	-0.18
12	36.84	36.82	-0.02
13	36.77	36.89	0.12
14	38.7	38.74	0.04
15	29.6	29.67	0.07
16	36	36.06	0.06
17	42.9	42.97	0.07

18	52.1	52.14	0.04
19	20.2	20.29	0.09
20	28.3	28.37	0.07
21	59.8	59.77	-0.03
22	30.9	30.94	0.04
23	113.3	113.13	-0.17
24	24.2	25.73	1.53
25	22.7	22.74	0.04
26	15.7	15.85	0.15
27	16.2	16.27	0.07
28	14.1	14.22	0.12
29	22.2	22.25	0.05
30	23.1	23.15	0.05
CO_2Me	51.7	51.72	0.02

Compound 2: ^{1}H NMR (300 MHz, $CDCl_3$) δ 5.32 (d, *J* = 5.7 Hz, 1H), 4.89 (d, *J* = 2.0 Hz, 1H), 4.78 (s, 1H), 3.65 (s, 3H), 2.26 – 1.72 (m, 9H), 1.71 (s, 3H), 1.68 – 1.28 (m, 10H), 1.25 (s, 3H), 1.01 (s, 3H), 0.98 – 0.91 (m, 1H), 0.89 (d, *J* = 6.5 Hz, 3H), 0.84 (s, 3H), 0.82 (d, *J* = 6.5 Hz, 3H), 0.77 (s, 3H), 0.75 (s, 3H). ^{13}C NMR (75 MHz, $CDCl_3$) δ 175.13, 146.25, 142.03, 119.22, 113.13, 59.77, 52.14, 51.72, 51.11, 42.97, 41.36, 40.51, 38.74, 36.89, 36.82, 36.08, 36.06, 30.94, 29.67, 29.64, 28.37, 25.73, 24.27, 23.15, 22.74, 22.25, 20.29, 19.52, 16.27, 15.85, 14.22. $C_{31}H_{51}O_2$: $[M+H]^+$ calc: 455.38836; found: 455.3882 m/z

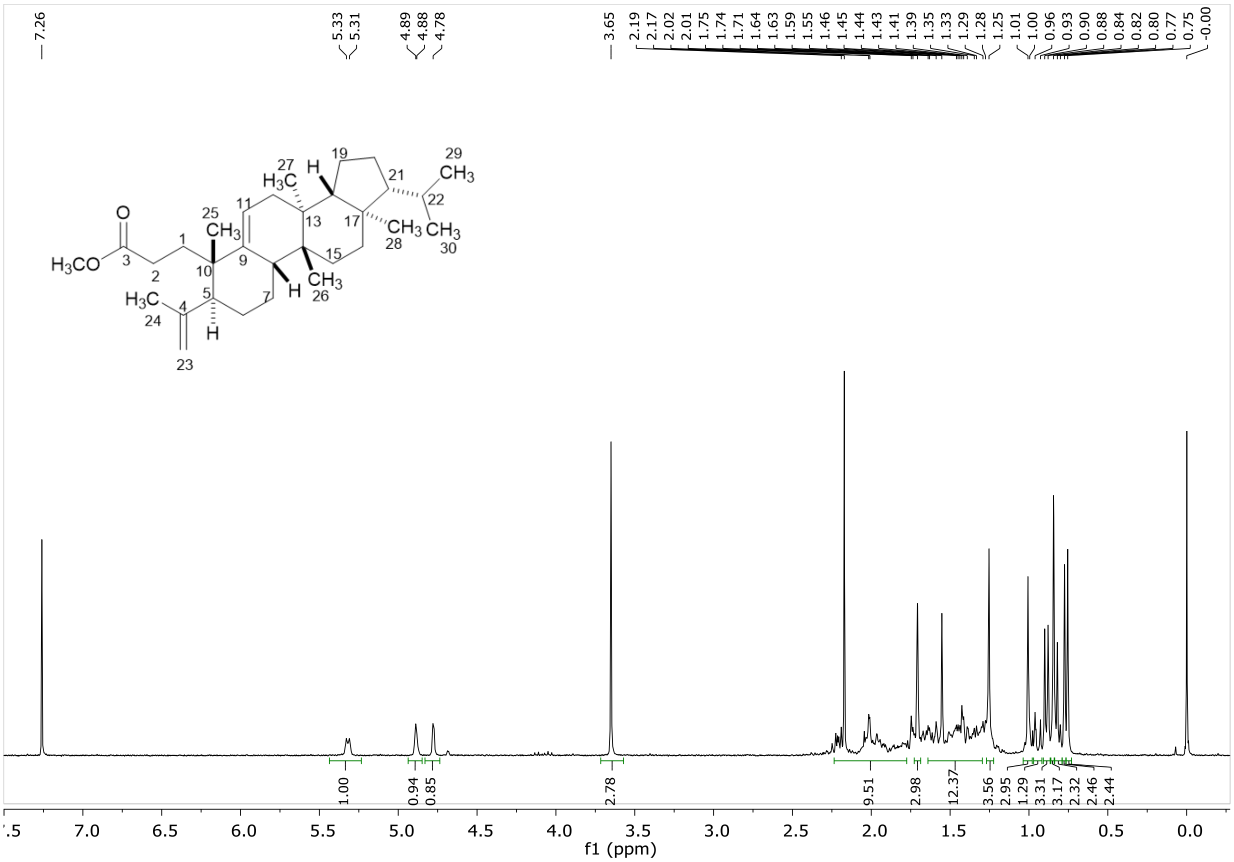

Figure S2.8: ^{1}H NMR spectrum (300 MHz, Chloroform-*d*) of compound 2

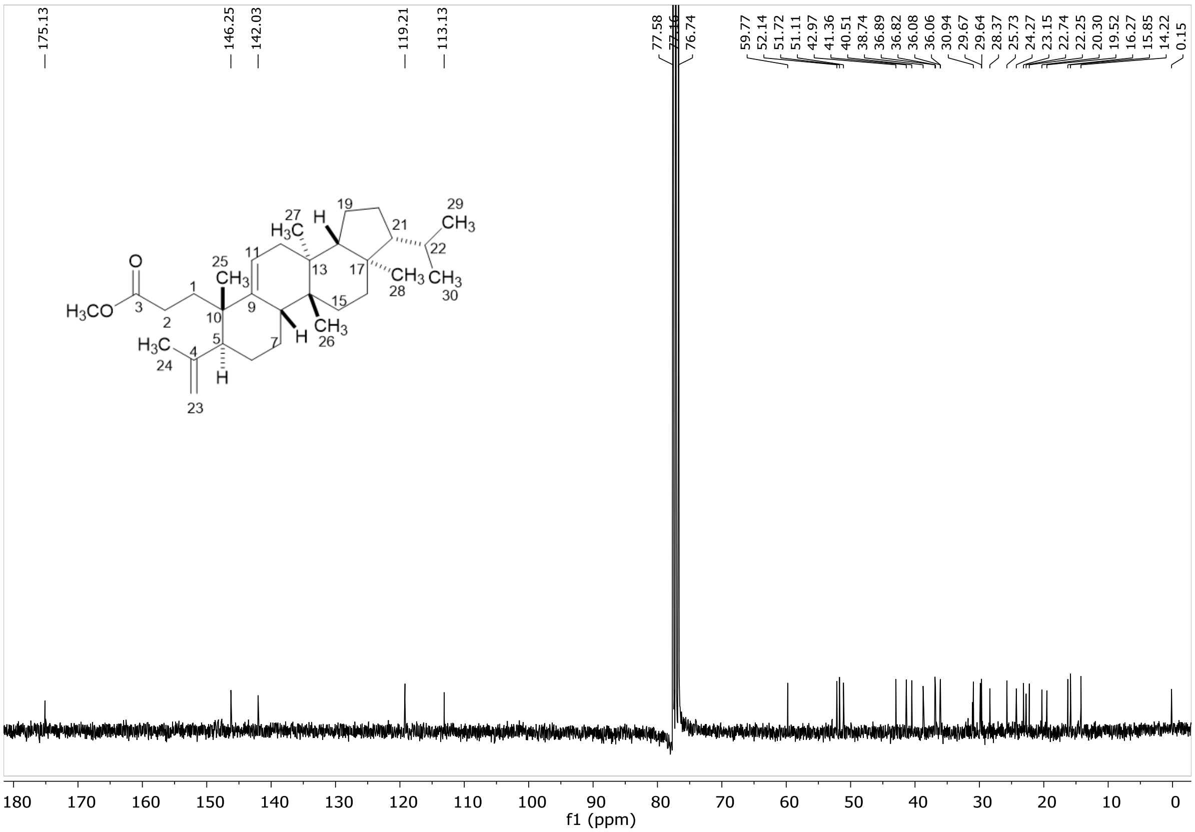

Figure S2.9: ^{13}C NMR spectrum (75 MHz, Chloroform-*d*) of compound 2

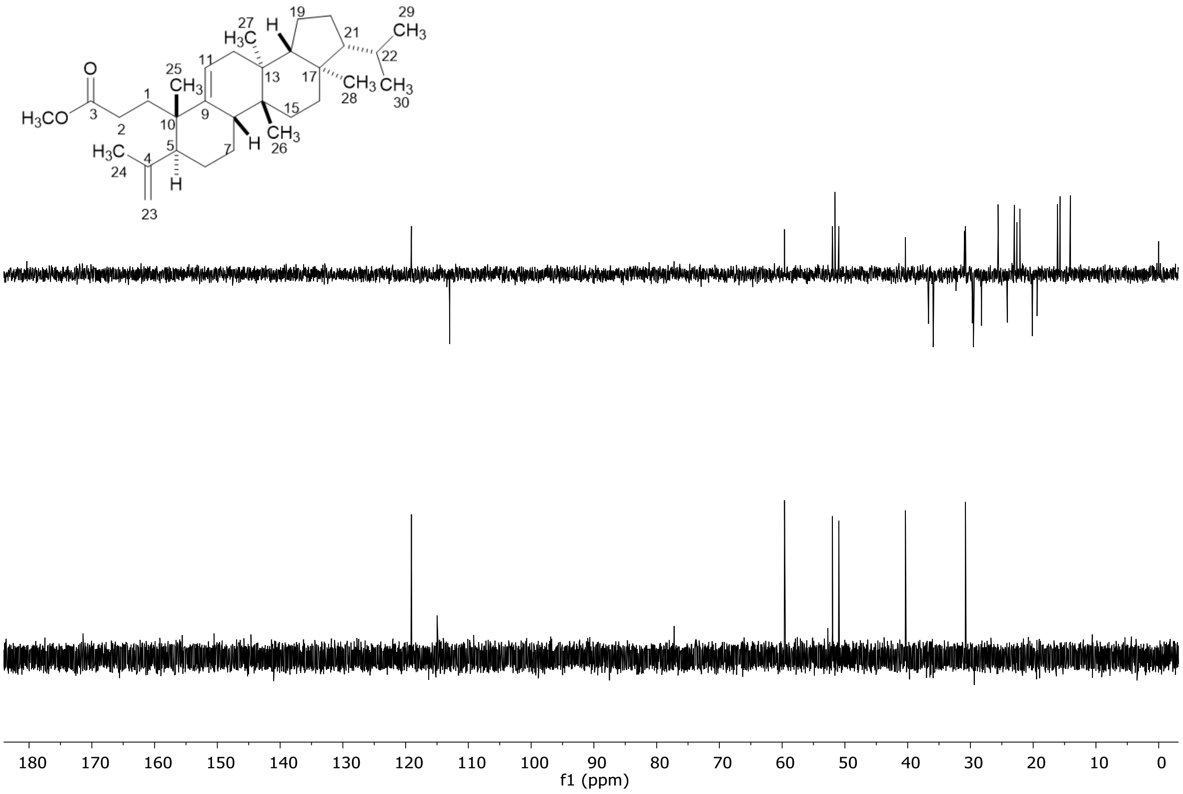

Figure S2.10: DEPT135 and DEPT90 spectra of compound 2

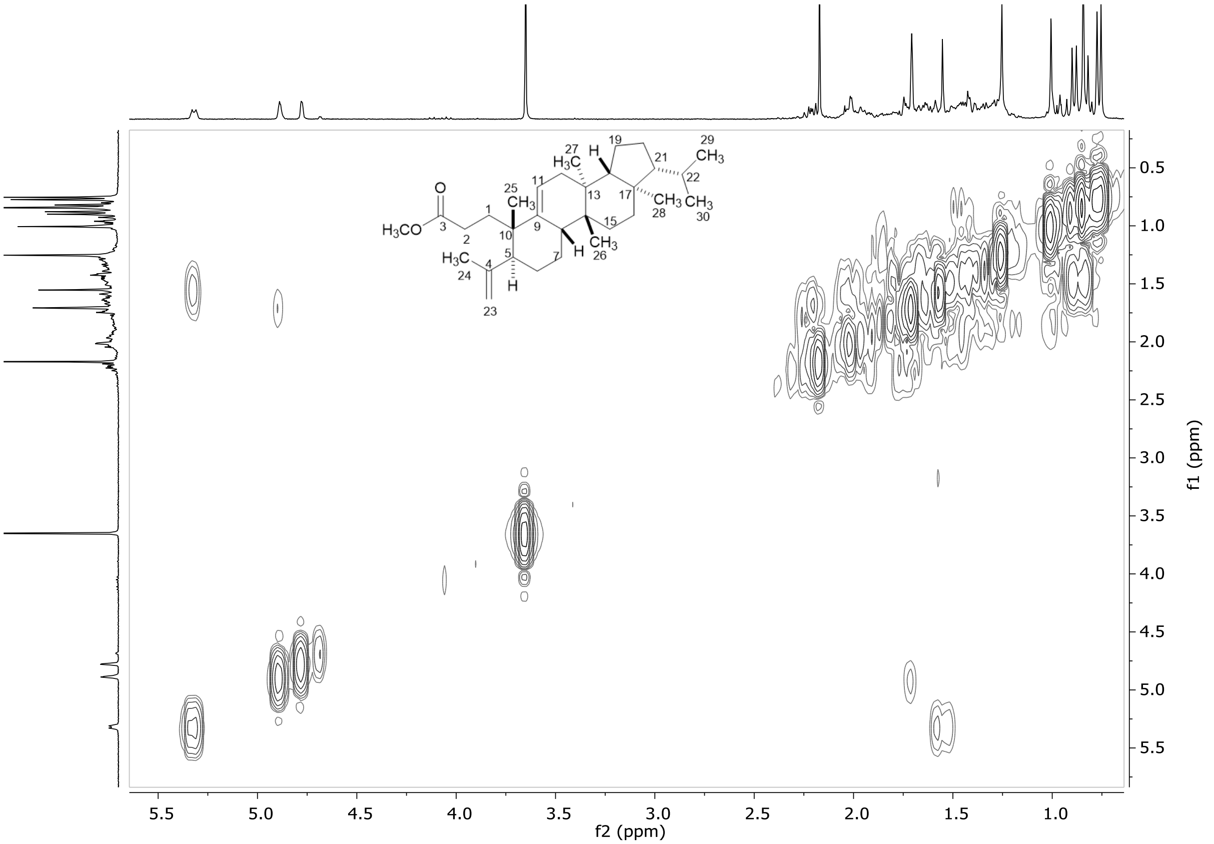

Figure S2.11: COSY spectrum of compound 2

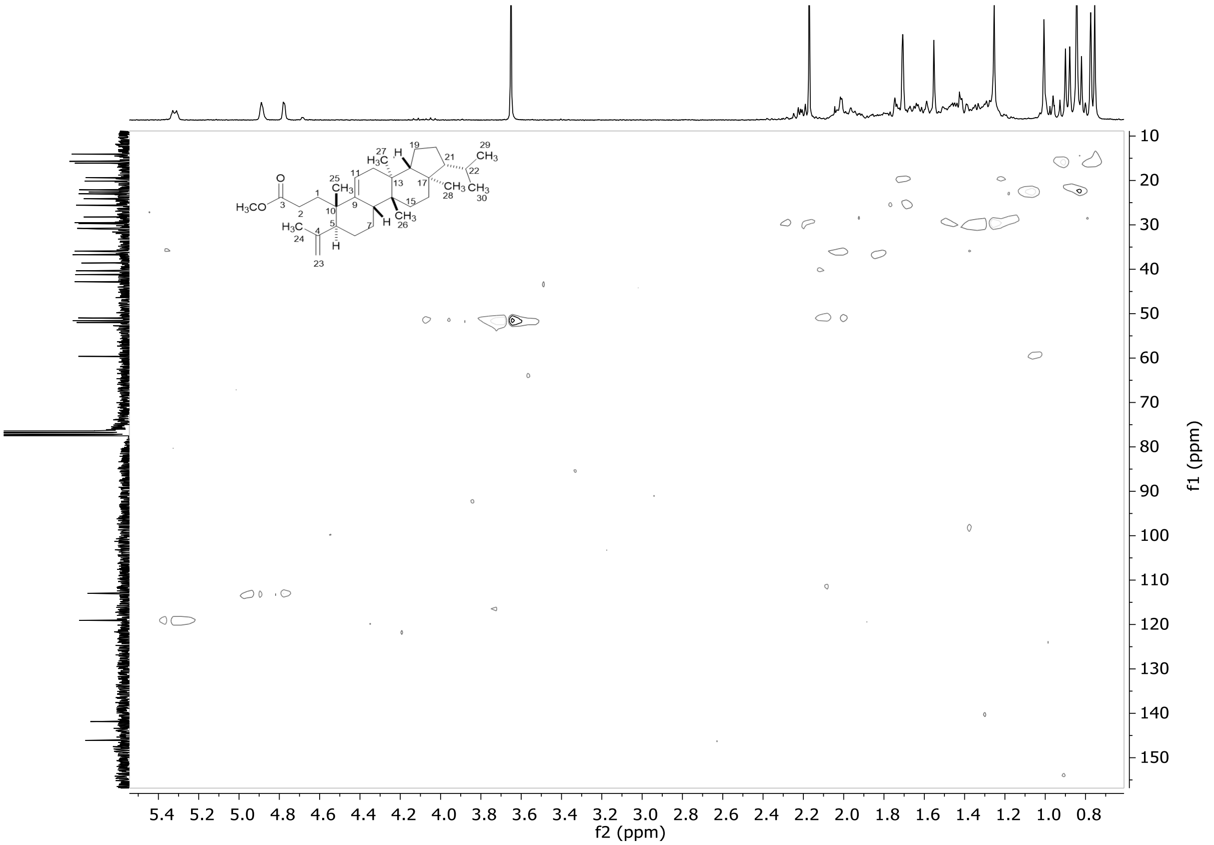

Figure S2.12: HSQC spectrum of compound 2

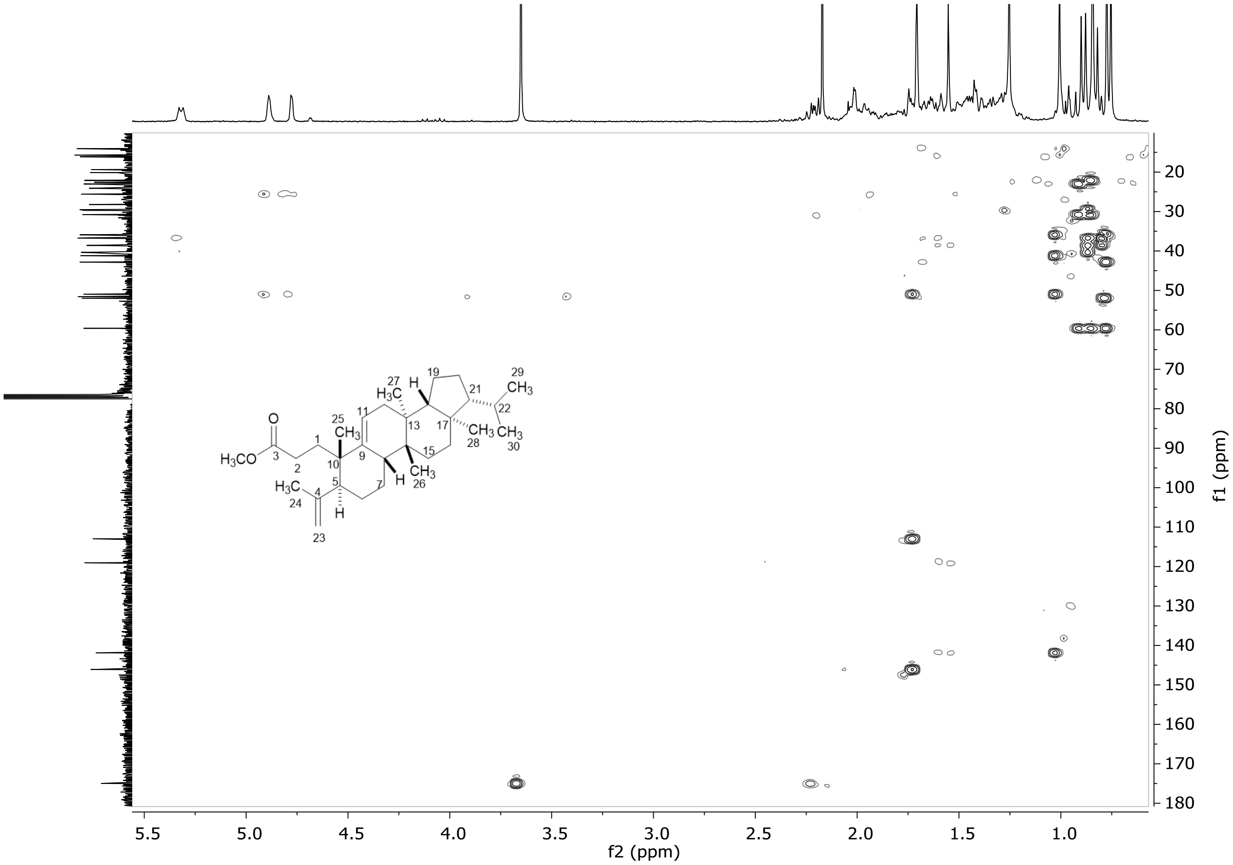

Figure S2.13: HMBC spectrum of compound 2

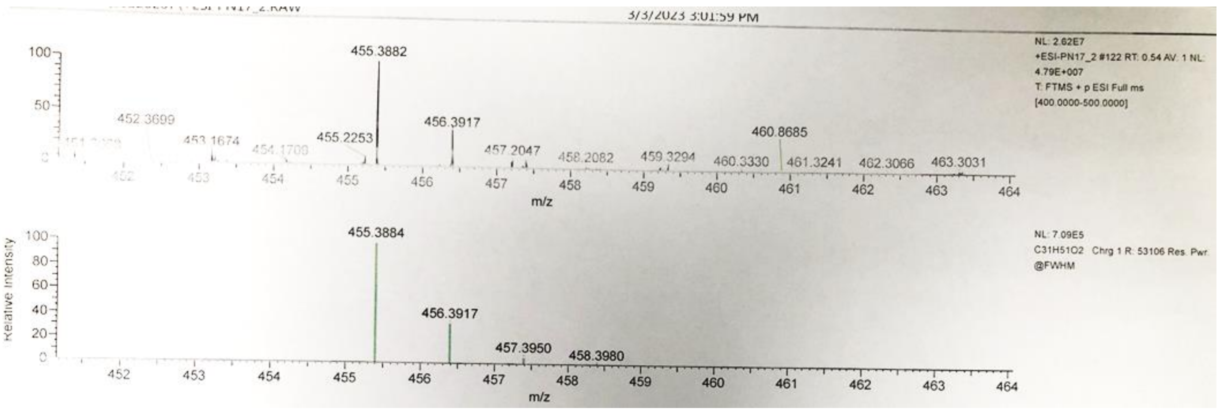

Figure S2.14: HRMS spectrum of compound 2

3. Compound 3 (2[4'-Hydroxyphenyl]-ethyl behenate])

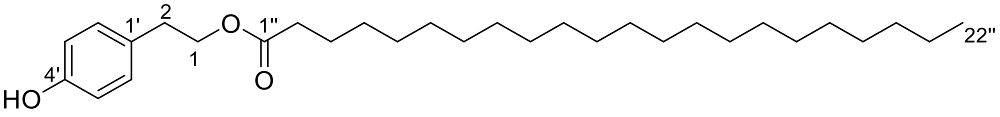

Proton	Reference: DOI 10.1055/s-2000-8570	Compound 3
	**^{1}H-NMR (500 MHz, $CDCl_3$) δ, ppm**	**^{1}H-NMR (600 MHz, $CDCl_3$) δ, ppm**
H-2', H-6'	7.08 (d, J=8.9 Hz)	7.08 (d, J = 8.5 Hz, 2H)
H-3', H-5'	6.75 (d, J=8.9 Hz)	6.76 (d, J = 8.5 Hz, 2H)
H-1	4.23 (t, J=7 Hz)	4.23 (t, J = 7.1 Hz, 2H)
H-2	2.87 (t, J=7 Hz)	2.86 (t, J = 7.1 Hz, 2H)
H-2"	2.31 (t, J=7.4 Hz)	2.27 (t, J = 7.6 Hz, 2H)
-OH	1.8	4.58 (s, 1H)
H-3"	1.57 (m)	1.58 (m, 2H)
-(CH2)-	1.25 (br s)	1.25 (s, 36H)
H-22"	0.88(t, J=6.5 Hz)	0.88 (t, J = 7.0 Hz, 3H)

carbon	Reference: DOI 10.1055/s-2000-8570	Compound 3	Δδ (ppm)
	**^{13}C-NMR (125 MHz, $CDCl_3$) δ, ppm**	**^{13}C-NMR (151 MHz, $CDCl_3$) δ, ppm**	
1"	173.8	174	0.2
4'	154.3	154.28	-0.02
1'	130.2	130.27	0.07
2',6'	130.1	130.21	0.11
3',5'	115.4	115.44	0.04
1	65.0	65.05	0.05
2	34.5	34.51	0.01
2"	34.4	34.43	0.03
3"	25.0	25.11	0.11
-(CH2)-	29.3-31.9	19.29-32.08	NA
21"	NA	22.85	NA
22"	14.1	14.28	0.18

Compound 3: ^{1}H NMR (600 MHz, $CDCl_3$) δ 7.08 (d, *J* = 8.4 Hz, 2H), 6.76 (d, *J* = 8.5 Hz, 2H), 4.58 (s, 1H), 4.23 (t, *J* = 7.1 Hz, 2H), 2.86 (t, *J* = 7.1 Hz, 2H), 2.27 (t, *J* = 7.6 Hz, 2H), 1.58 (t, *J* = 7.2 Hz, 2H), 1.25 (s, 36H), 0.88 (t, J = 7.0 Hz, 3H). ^{13}C NMR (151 MHz, $CDCl_3$) δ 174.00, 154.28, 130.24, 115.44, 65.05, 34.51, 34.43, 32.08, 29.86, 29.82, 29.77, 29.62, 29.52, 29.43, 29.29, 25.11, 22.85, 14.28. $C_{30}H_{52}O_3{}^{23}Na$: $[M+Na]^+$ calc: 483.38087; found: 483.3807 m/z

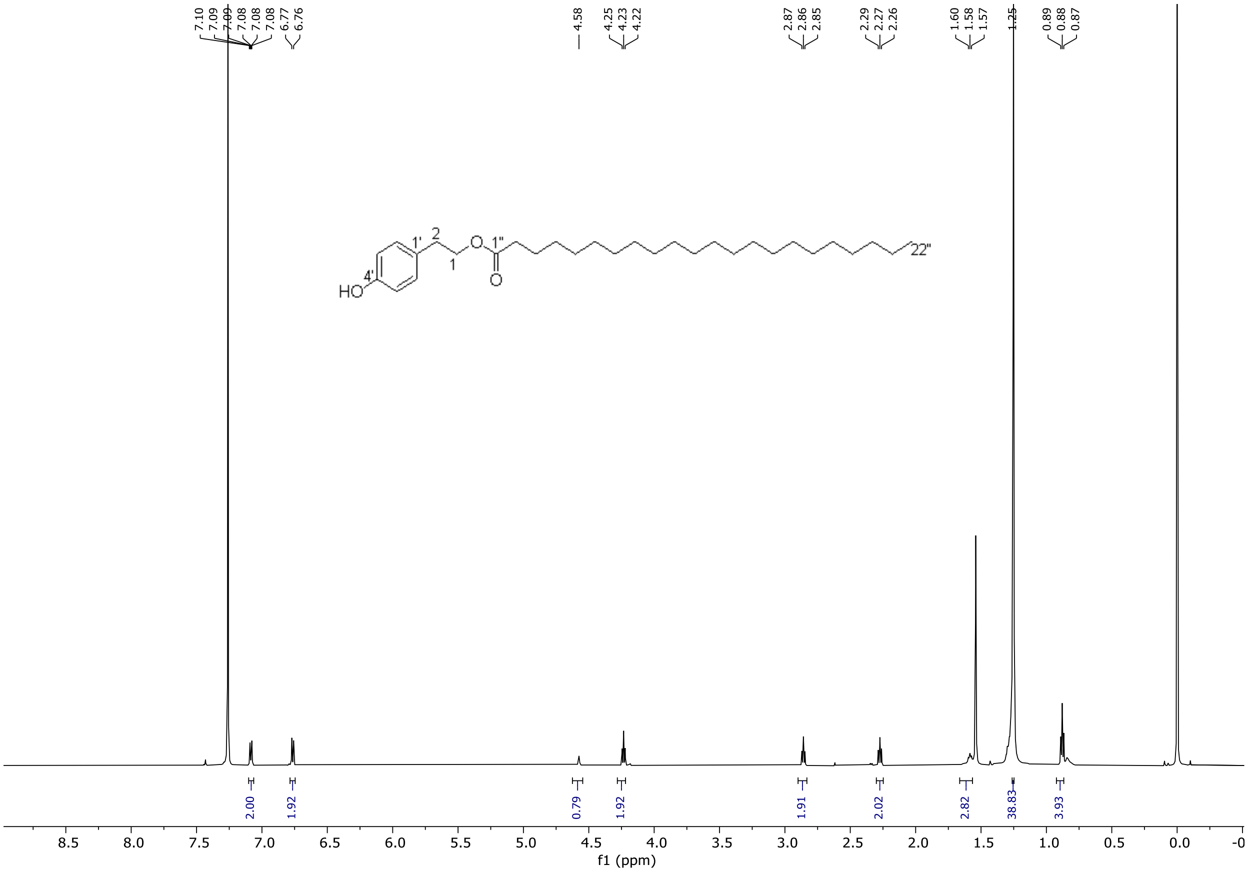

Figure S2.15: ^{1}H NMR spectrum (600 MHz, Chloroform-d) of compound 3

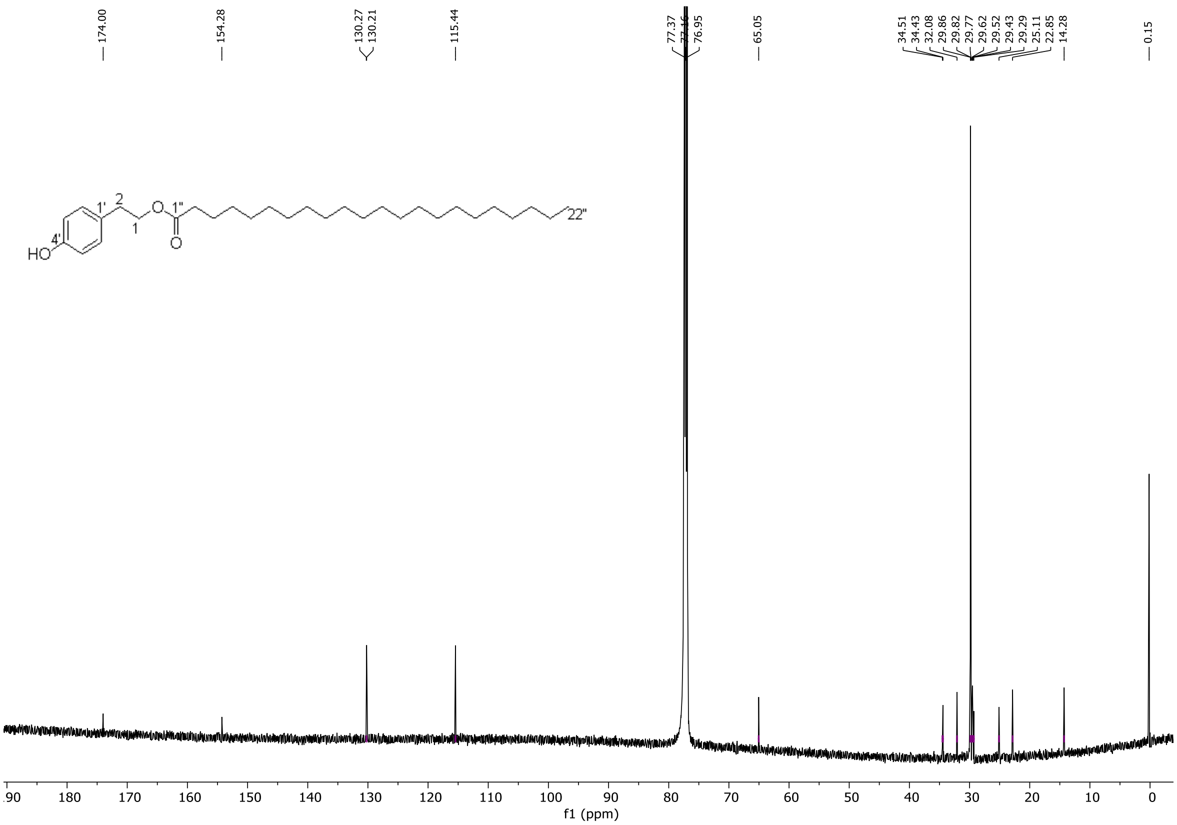

Figure S2.16: ^{13}C NMR spectrum (151 MHz, Chloroform-d) of compound 3

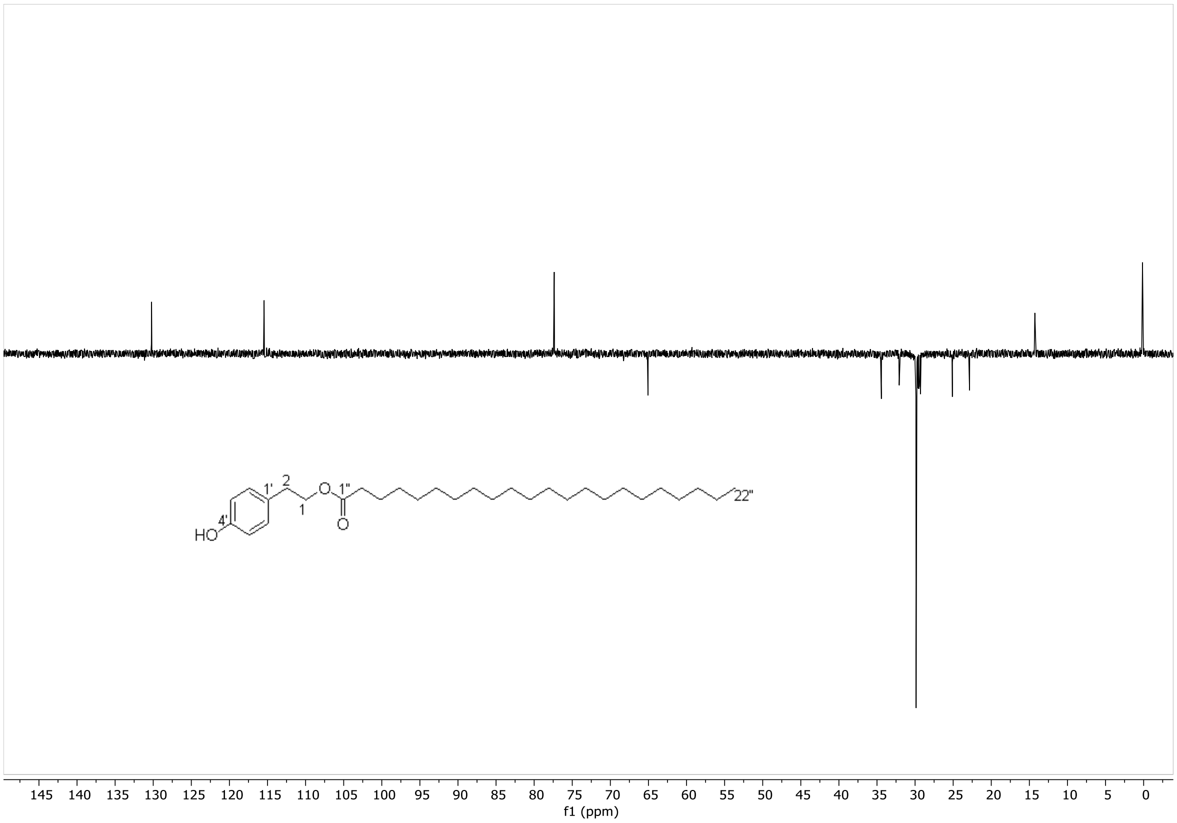

Figure S2.17: DEPT135 spectrum of compound 3

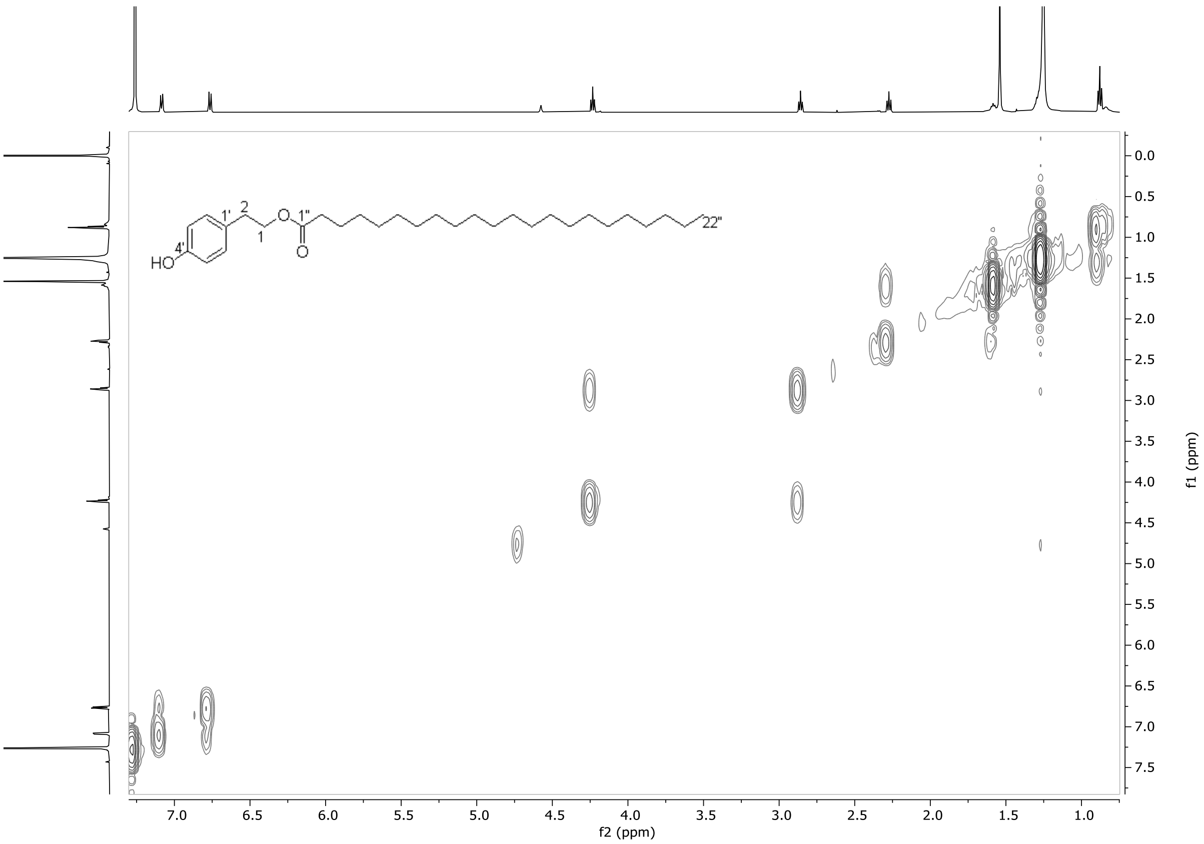

Figure S2.18: COSY spectrum of compound 3

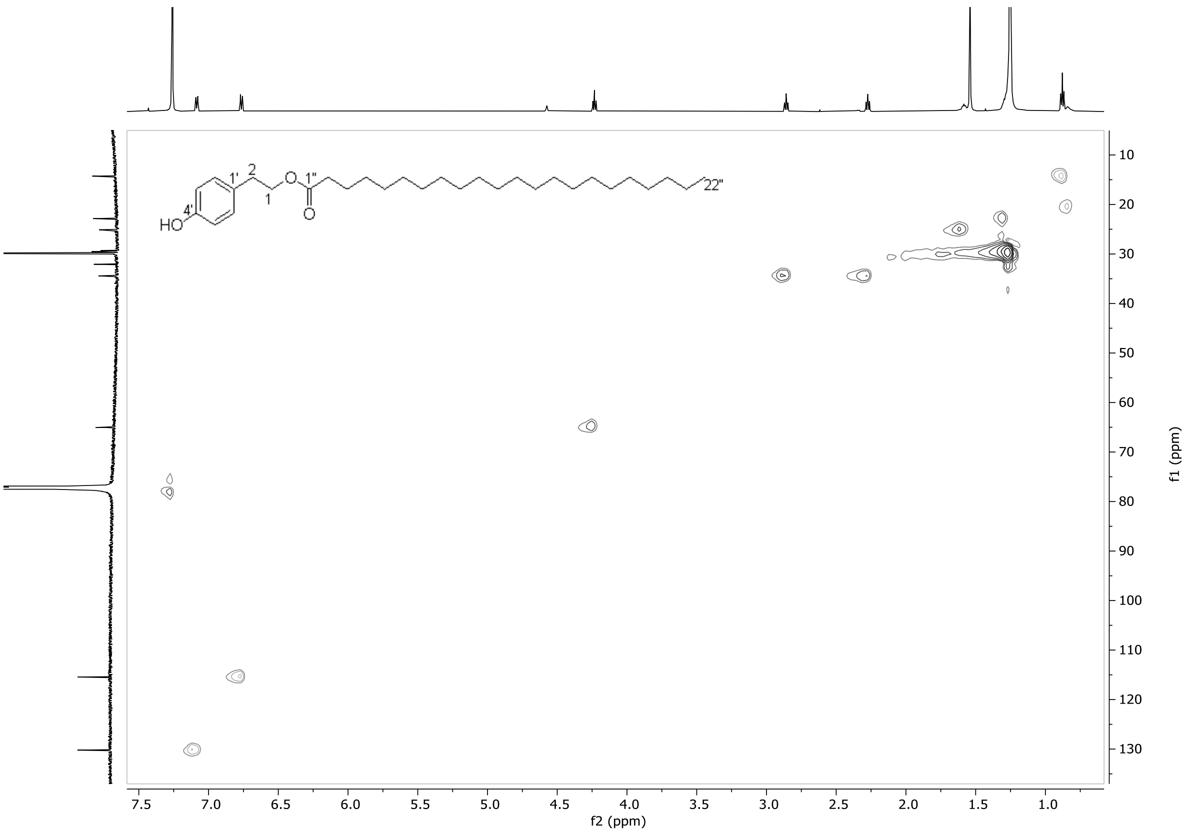

Figure S2.19: HSQC spectrum of compound 3

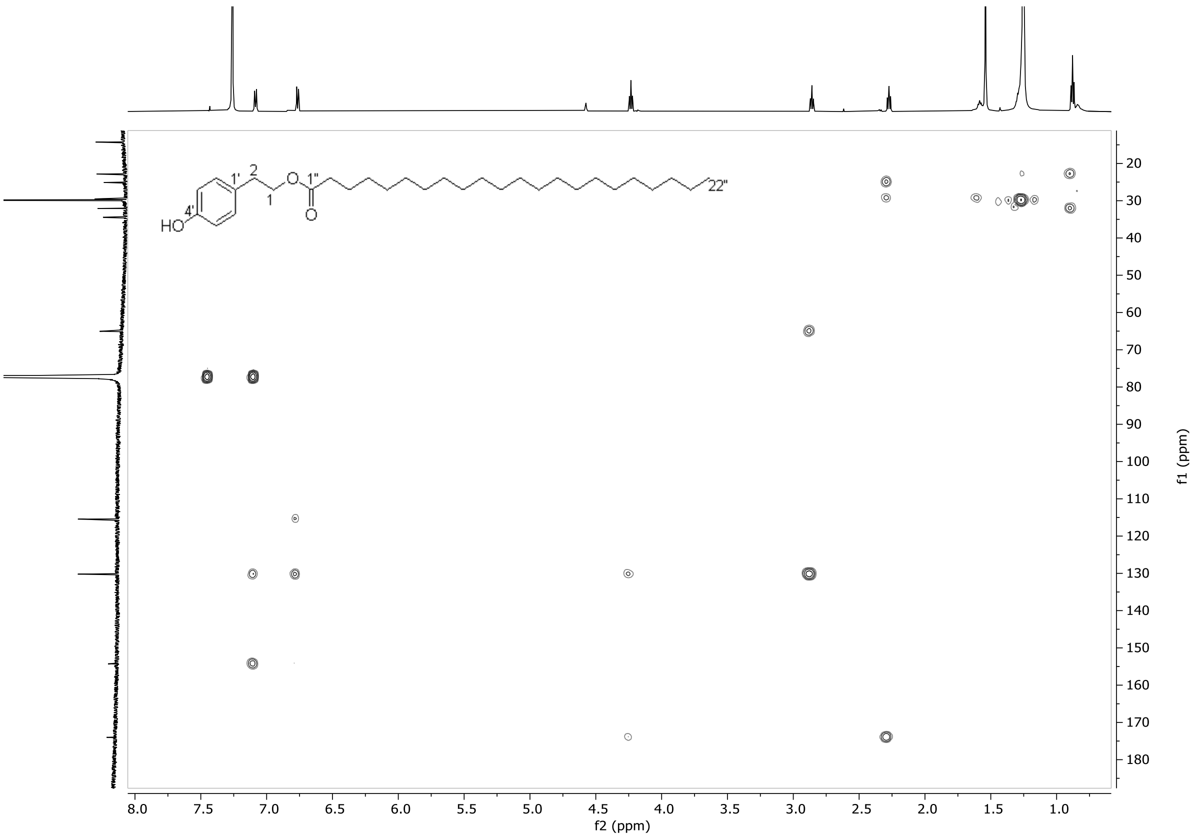

Figure S2.20: HMBC spectrum of compound 3

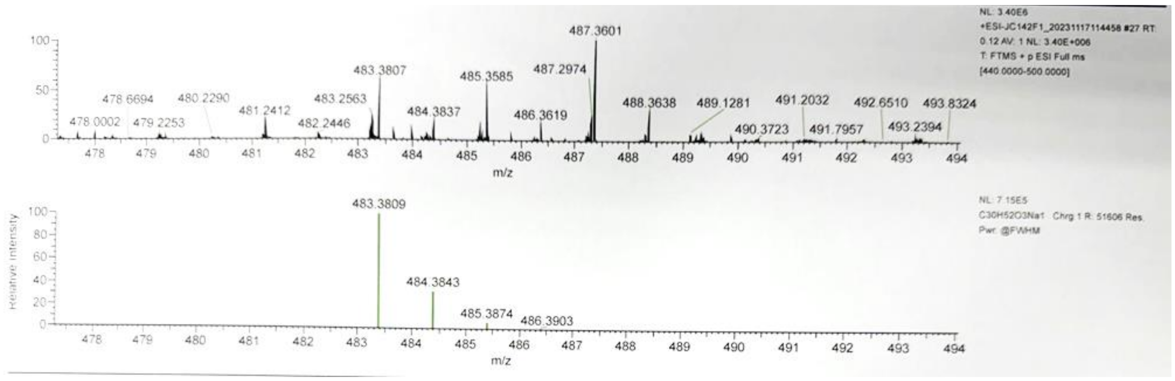

Figure S2.21: HRMS spectrum of compound 3

4. Compound 4 (3,4-seco-8βH-fernadienoic acid)

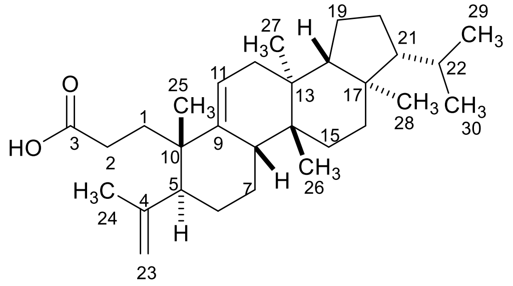

Proton	Reference: DOI 10.1016/0031-9422(95)00721-0	Compound 4
	^{1}H-NMR (400 MHz, $CDCl_3$) δ, ppm	^{1}H-NMR (600 MHz, $CDCl_3$) δ, ppm
H-1, H-5	H1 (1.74, 1.98), H5 (2.02)	2.07 – 1.93 (m, 3H)
H-2	2.50	2.33 – 2.18 (m, 2H)
H-6	1.47, 1.97	
H-7	1.44, 1.63	
H-8	2.03	
H-11	5.33 (ddd, J=5.8, 2.0, 2.0 Hz)	5.33 (d, J = 5.7 Hz, 1H)
H-12	1.79, 1.56	
H-15	1.42, 1.32	
H-16	1.66, 1.38	
H-18	1.61	
H-19	1.27, 1.37	
H-20	1.22, 1.85	1.89 – 1.80 (m, 1H),
H-21	0.99	1.01 – 0.94 (m, 1H)
H-22	1.43	
H-23	4.78 (d, J=1.8), 4.90 (t, J=1.8)	4.78 (s, 1H), 4.89 (s, 1H)
H-24	1.71	1.71 (s, 3H)
H-25	1.02	1.02 (s, 3H)
H-26	0.78	0.78 (s, 3H)
H-27	0.85	0.85 (s, 3H)
H-28	0.76	0.75 (s, 3H)
H-29	0.89 (d, J=6.5 Hz)	0.89 (d, J = 6.5 Hz, 3H)
H-30	0.83 (d, J=6.5 Hz)	0.83 (d, J = 6.5 Hz, 3H)

carbon	Reference: DOI 10.1016/0031-9422(95)00721-0	Compound 4	Δδ (ppm)
	^{13}C-NMR (125 MHz, $CDCl_3$) δ, ppm	^{13}C-NMR (151 MHz, $CDCl_3$) δ, ppm	
1	35.6	36.04	0.44
2	29.5	28.85	-0.65
3	180.7	176.40	-4.3
4	146.0	146.20	0.2
5	51.0	51.22	0.22
6	24.1	24.25	0.15
7	19.4	19.51	0.11
8	40.4	40.51	0.11
9	141.7	141.89	0.19
10	41.2	41.35	0.15
11	119.3	119.40	0.1
12	36.7	36.81	0.08
13	36.7	36.88	0.22
14	38.6	38.74	0.14
15	29.5	29.64	0.14
16	35.9	35.81	-0.09
17	42.8	42.96	0.16
18	52.0	52.12	0.12
19	20.2	20.29	0.09
20	28.2	28.37	0.17
21	59.6	59.76	0.16
22	30.8	30.94	0.14
23	113.1	113.19	0.09
24	25.6	25.72	0.12
25	22.6	22.72	0.12
26	15.7	15.87	0.17
27	16.1	16.27	0.17
28	14.1	14.21	0.11
29	22.1	22.25	0.15

30	23.0	23.15	0.15

Compound 4: ^1H NMR (600 MHz, $CDCl_3$) δ 5.33 (d, *J* = 5.7 Hz, 1H), 4.89 (s, 1H), 4.78 (s, 1H), 2.33 – 2.18 (m, 2H), 2.07 – 1.93 (m, 3H), 1.89 – 1.80 (m, 1H), 1.71 (s, 3H), 1.02 (s, 3H), 1.67-1.20 (m, 16H), 1.01 – 0.94 (m, 1H), 0.89 (d, *J* = 6.5 Hz, 3H), 0.85 (s, 3H), 0.83 (d, *J* = 6.5 Hz, 3H), 0.78 (s, 3H), 0.75 (s, 3H). ^{13}C NMR (151 MHz, $CDCl_3$) δ 176.40, 146.20, 141.89, 119.40, 113.19, 59.76, 52.12, 51.22, 42.96, 41.35, 40.51, 38.74, 36.88, 36.81, 36.04, 35.81, 30.94, 29.64, 28.85, 28.37, 25.72, 24.25, 23.15, 22.72, 22.25, 20.29, 19.51, 16.27, 15.87, 14.2. $C_{30}H_{49}O_2$: $[M+H]^+$ calc: 441.37271; found: 441.3748 m/z

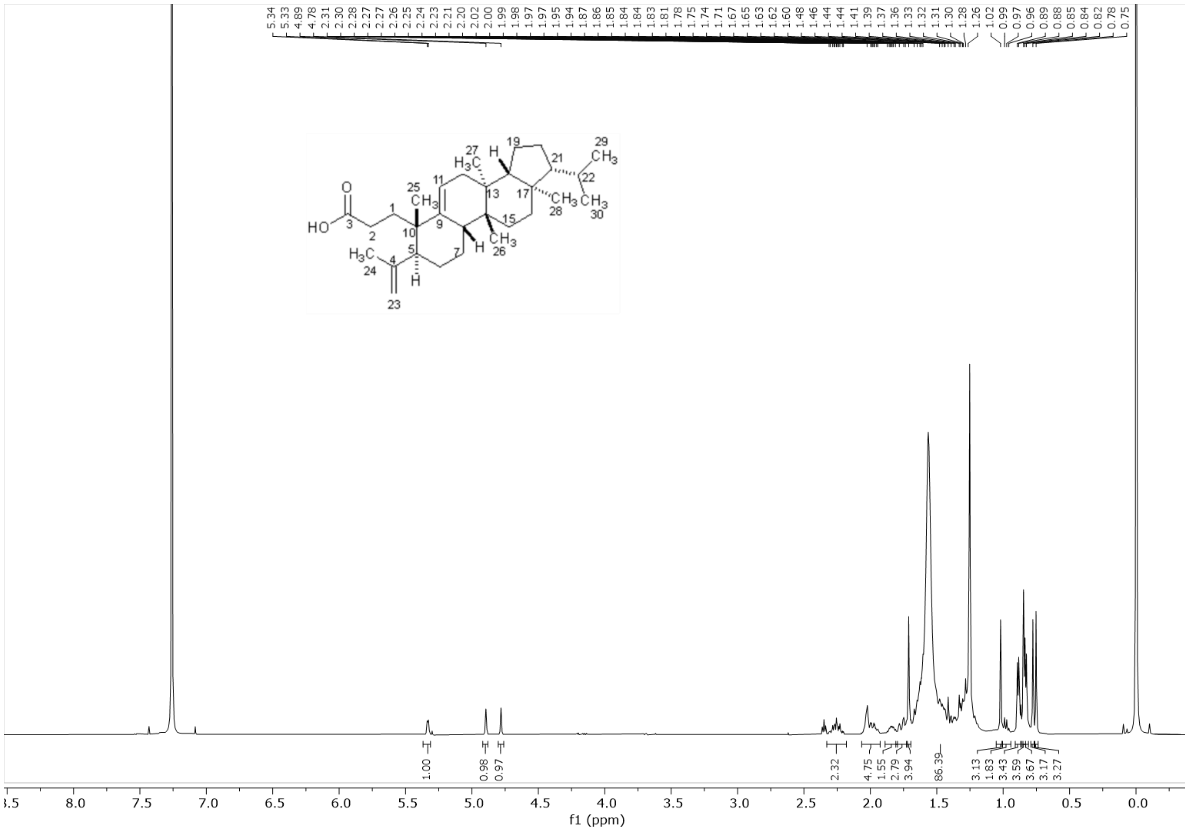

Figure S2.22: ^1H NMR spectrum (600 MHz, Chloroform-d) of compound 4

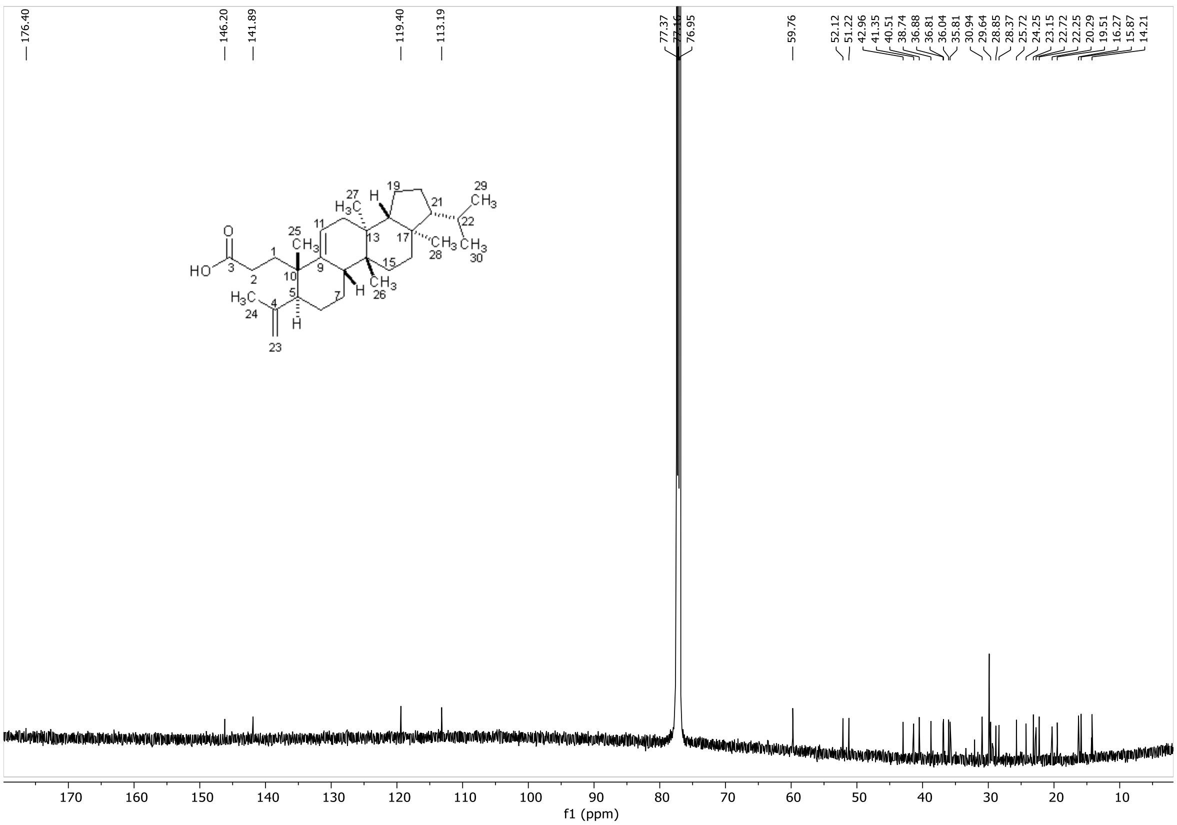

Figure S2.23: ^{13}C NMR spectrum (600 MHz, Chloroform-d) of compound 4

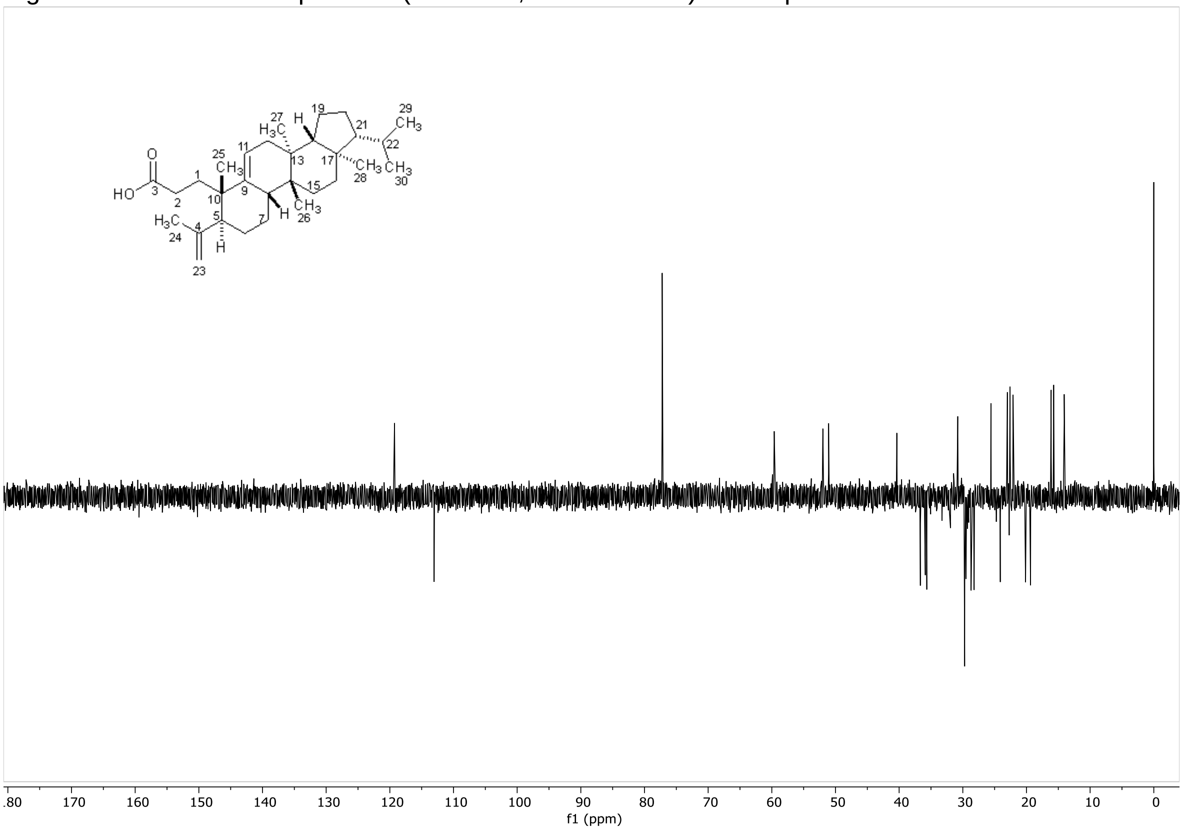

Figure S2.24: DEPT135 spectrum of compound 4

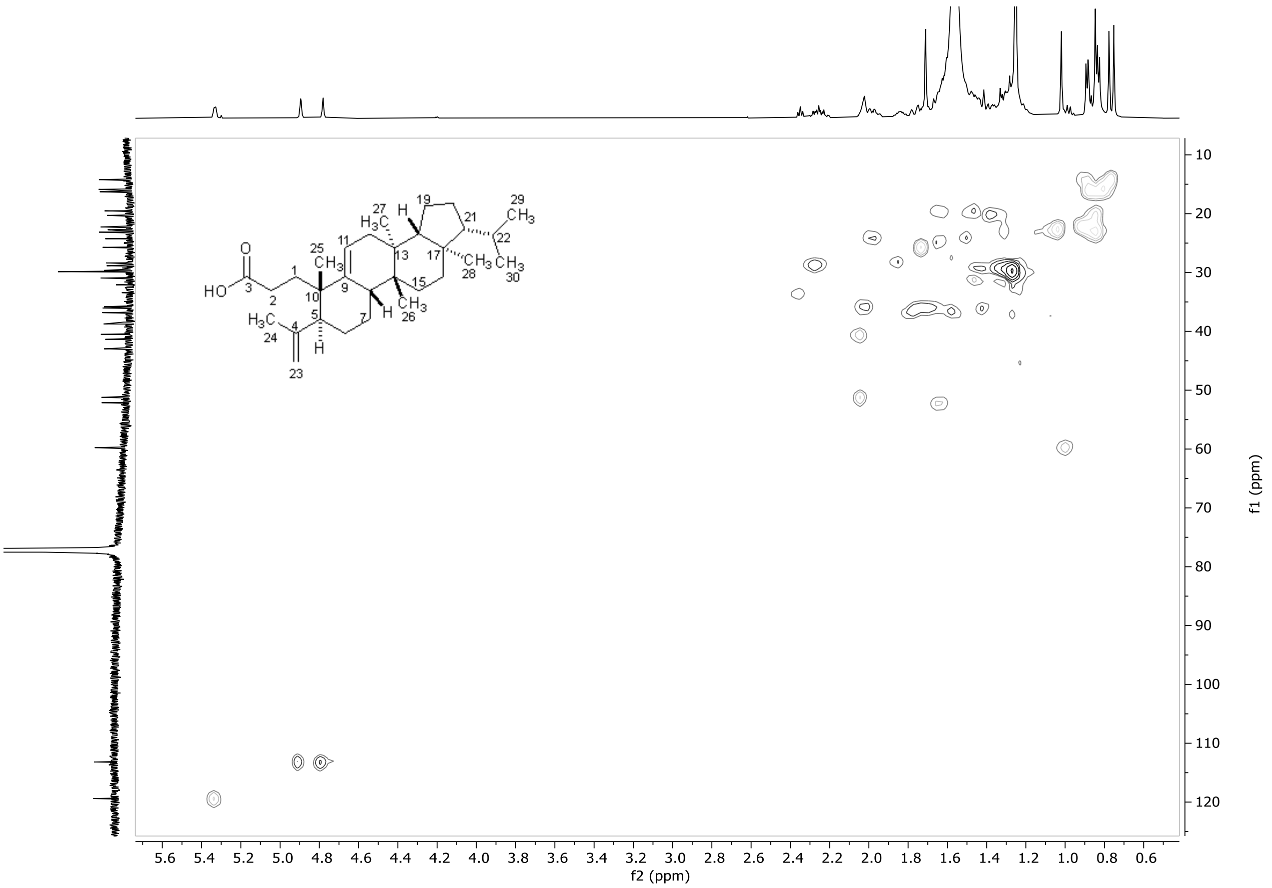

Figure S2.25: HSQC spectrum of compound 4

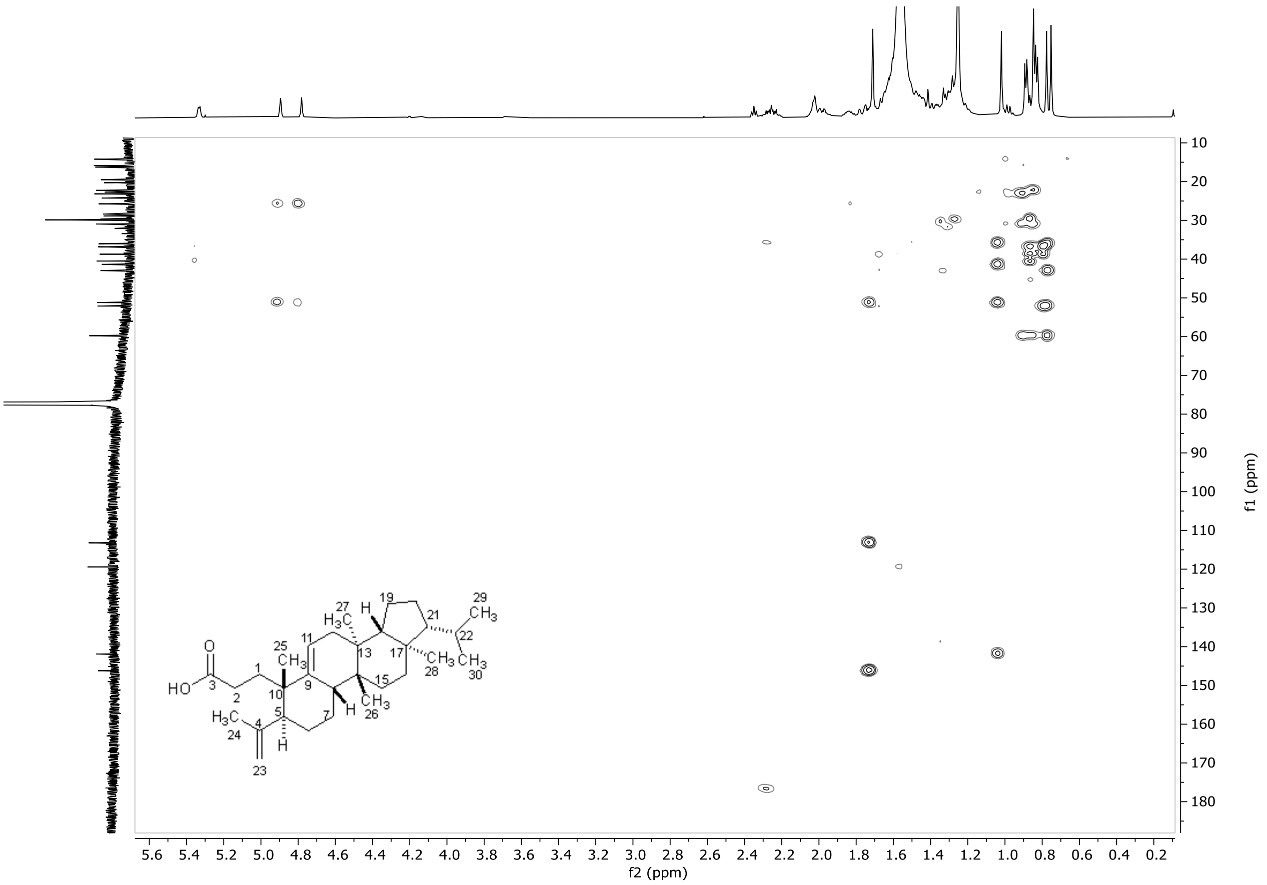

Figure S2.26: HMBC spectrum of compound 4

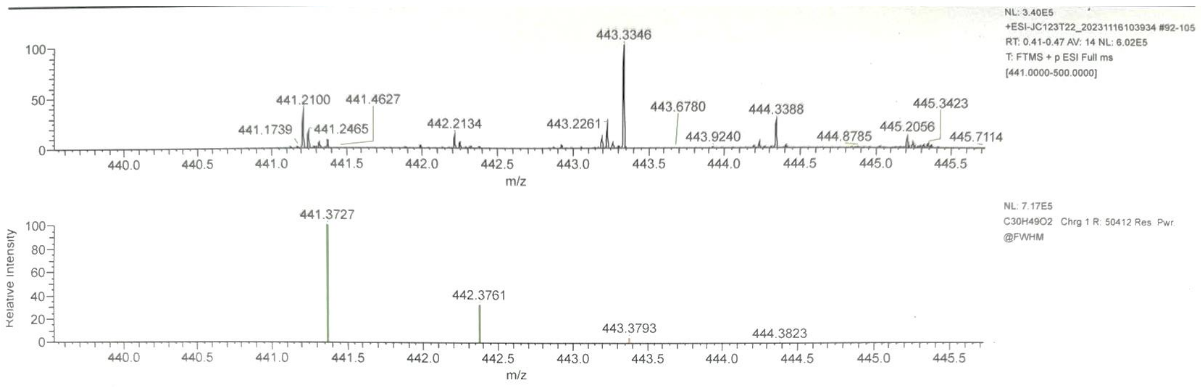

Figure S2.27: HRMS spectrum of compound 4

5. Compound 5 (Lutein)

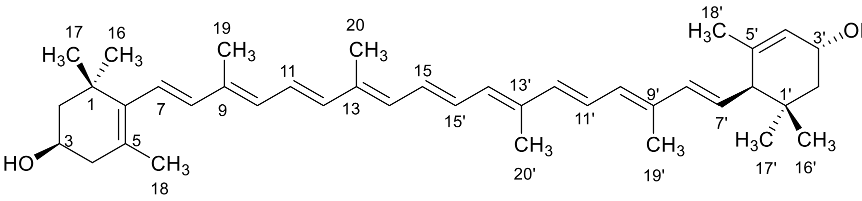

Proton	Reference: DOI 10.3390/molecules26020503<br>^{1}H-NMR (500 MHz, $CDCl_3$) δ, ppm	Compound 5<br>^{1}H-NMR (300 MHz, $CDCl_3$) δ, ppm
H-11, H-15, H-11’, H-15’	6.69 - 6.56 (br m, 4H)	6.72 – 6.54 (m, 4H)
H-12, H-12’	6.35 (d, J = 14.8 Hz, 2H)	6.36 (d, J = 14.9 Hz, 2H)
H-14, H-14’	6.28 - 6.22 (br m, 2H)	6.25 (m, 2H)
H-10, H-8’, H-10’<br>H-7, H-8	6.18 - 6.13 (br m, 3H)<br>6.12 (m, 2H)	6.14 (m, 5H)
H-4’	5.54 (s, 1H)	5.54 (s, 1H)
H-7’	5.43 (dd, J = 15.3, 9.8 Hz, 1H)	5.43 (dd, J = 15.5, 9.9 Hz, 1H)
H-3’	4.25 (s, 1H)	4.25 (s, 1H)
H-3	4.05 - 3.95 (br m, 1H)	4.00 (m, 1H)
H-4, H-6’	2.44 - 2.34 (br m, 2H)	2.47 – 2.33 (m, 2H)
H-4	2.04 (dd, J = 17.0, 9.6 Hz, 1H)	2.13 – 2.01 (m, 1H)
H-19, H-20, H-19’, H-20’	1.97 (s, 12H)	1.97 (s, 9H)<br>1.91 (s, 3H)
H-2	1.84 (dd, J = 13.1, 5.8 Hz, 1H)<br>1.83 – 1.76 (br m, 1H)	1.87 – 1.76 (m, 2H)
H-18	1.74 (s, 3H)	1.73 (s, 3H)
H-18’	1.62 (s, 3H)	1.62 (s, 3H)
H-2'	1.48 (t, J = 11.9 Hz, 1H)	1.47 (t, J = 11.9 Hz, 1H)
H-2’	1.36 (dd, J = 13.1, 6.8 Hz, 1H)	1.36 (dd, J = 13.2, 6.9 Hz, 1H)
H-16, H17, H-16’, H17'	1.07 (s, 6H)<br>0.85 (s, 6H)	1.07 (s, 6H)<br>1.00 (s, 3H)<br>0.85 (s, 3H)

Compound 5: ^{1}H NMR (300 MHz, $CDCl_3$) δ 6.72 – 6.54 (m, 4H, H-11, H-15, H-11’, H-15’), 6.36 (d, *J* = 14.9 Hz, 2H, H-12, H-12’), 6.25 (m, 2H, H-14, H-14’), 6.14 (m, 5H, H-7, H-8, H-8’, H-10, H-10’), 5.54 (s, 1H, H-4’), 5.43 (dd, *J* = 15.5, 9.9 Hz, 1H, H-7’), 4.25 (s, 1H, H-3’), 4.00 (m, 1H, H-3), 2.47 – 2.33 (m, 2H, H-4, H-6’), 2.13 – 2.01 (m, 1H, H-4), 1.97 (s, 9H, H-19, H-20, H-19’), 1.91 (s, 3H, H-20’), 1.87 – 1.76 (m, 2H, H-2), 1.73 (s, 3H, H-18), 1.62 (s, 3H, H-18’), 1.47 (t, *J* = 11.9 Hz, 1H, H-2’), 1.36 (dd, *J* = 13.2, 6.9 Hz, 1H, H-2’), 1.07 (s, 6H, H-16, H17), 1.00 (s, 3H, H-16’), 0.85 (s, 3H, H-17’). $C_{40}H_{56}O_2$: $M^{•+}$ calc: 568.42803; found: 568.4279 m/z.

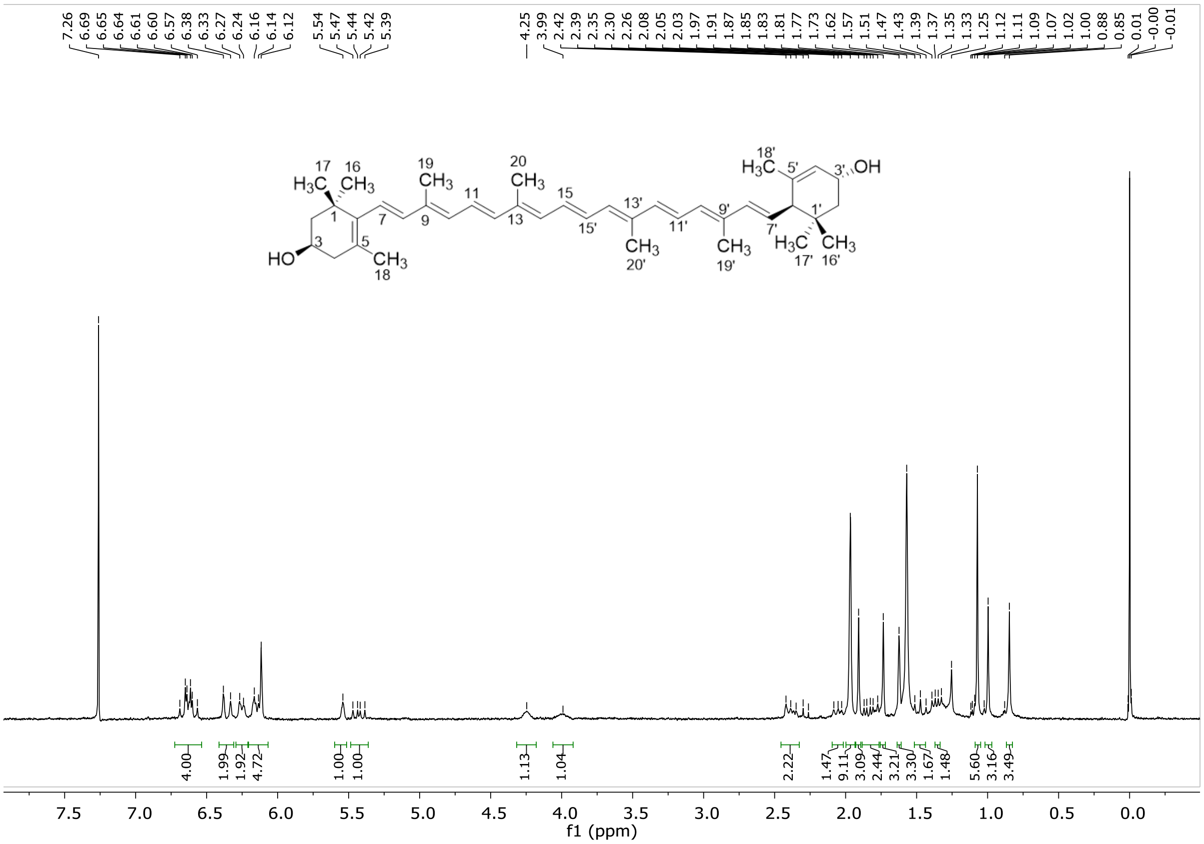

Figure S2.28: ^{1}H NMR spectrum (300 MHz, Chloroform-*d*) of compound 5 (lutein)

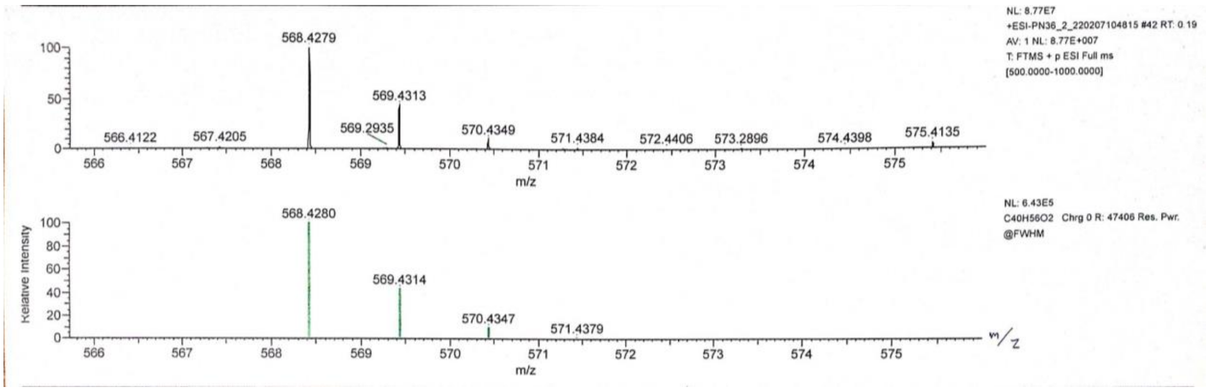

Figure S2.29: HRMS spectrum of compound 5 (lutein)

This work

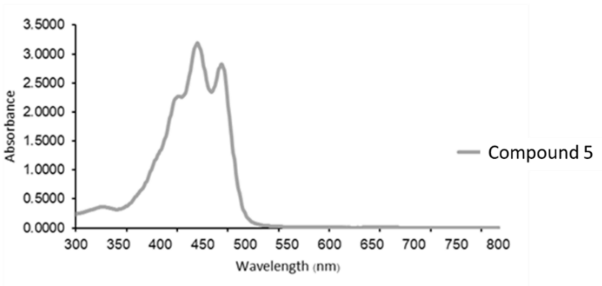

Reference

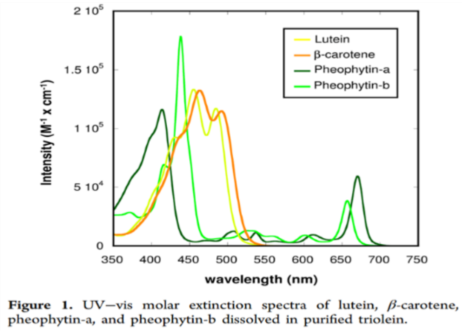

Figure S2.30: UV-Vis spectrum of compound 5 in MeOH, as compared to the previously reported spectrum *(J. Agric. Food Chem. 2014, 62, 38, 9317–9325 (https://doi.org/10.1021/jf503818k))*

6. Compound 6 (Glyceryl palmitate)

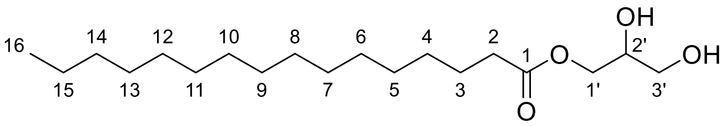

Proton	Reference: DOI 10.1021/acs.jafc.9b06655	Compound 6
	**^{1}H-NMR (400 MHz, $CDCl_3$) δ, ppm**	**^{1}H-NMR (300 MHz, $CDCl_3$) δ, ppm**
H-1'	4.21 (dd, J = 4.6 and 11.7 Hz, 1H)	4.21 (dd, J = 11.7, 4.7 Hz, 1H)
H-1'	4.15 (dd, J = 6.1 and 11.7 Hz, 1H)	4.15 (dd, J = 11.7, 6.0 Hz, 1H)
H-2'	3.93 (p, J = 5.7 Hz, 1H)	3.93 (p, J = 5.3 Hz, 1H)
H-3'	3.65 (m, 2H)	3.70 (dd, J = 11.5, 4.0 Hz, 1H)
		3.60 (dd, J = 11.4, 5.8 Hz, 1H)
H-2	2.35 (t, J = 7.5 Hz, 2H,)	2.35 (t, J = 7.5 Hz, 2H)
H-3	1.63 (p, J = 7.5 Hz, 2H)	1.62 (m, 2H)
H-4 to H-15	1.26 (m, 24H)	1.25 (m, 24H)
H-16	0.88 (t, J = 6.8 Hz, 3H)	1.04 – 0.81 (m, 3H).

carbon	Reference: DOI 10.1021/acs.jafc.9b06655	Compound 6	Δδ (ppm)
	**13C-NMR (100 MHz, $CDCl_3$) δ, ppm**	**13C-NMR (75 MHz, $CDCl_3$) δ, ppm**	
C-1	174.3	174.5	0.2
C-2'	70.3	70.43	0.13
C-1'	65.2	65.32	0.12
C-3'	63.3	63.48	0.18
C-2	34.1	34.31	0.21
C-14	31.9	32.08	0.18
C-4−C-13	29.7	29.84	0.14
	29.6	29.75	0.15
	29.4	29.6	0.2
	29.3	29.51	0.21

	29.2<br>29.1	29.4<br>29.28	0.2<br>0.18
C-3	24.9	25.07	0.17
C-15	22.7	22.85	0.15
C-16	14.1	14.28	0.18

Compound 6: ^{1}H NMR (300 MHz, $CDCl_3$) δ 4.21 (dd, *J* = 11.7, 4.7 Hz, 1H), 4.15 (dd, *J* = 11.7, 6.0 Hz, 1H), 3.93 (p, *J* = 5.3 Hz, 1H), 3.70 (dd, *J* = 11.5, 4.0 Hz, 1H), 3.60 (dd, *J* = 11.4, 5.8 Hz, 1H), 2.35 (t, *J* = 7.5 Hz, 2H), 1.62 (m, 2H), 1.25 (m, 24H), 1.04 – 0.81 (m, 3H). ^{13}C NMR (75 MHz, $CDCl_3$) δ 174.50, 70.43, 65.32, 63.48, 34.31, 32.08, 29.84, 29.75, 29.60, 29.51, 29.40, 29.28, 25.07, 22.85, 14.28. $C_{19}H_{39}O_4$: $[M+H]^+$ calc: 331.28429; found: 331.2843 m/z

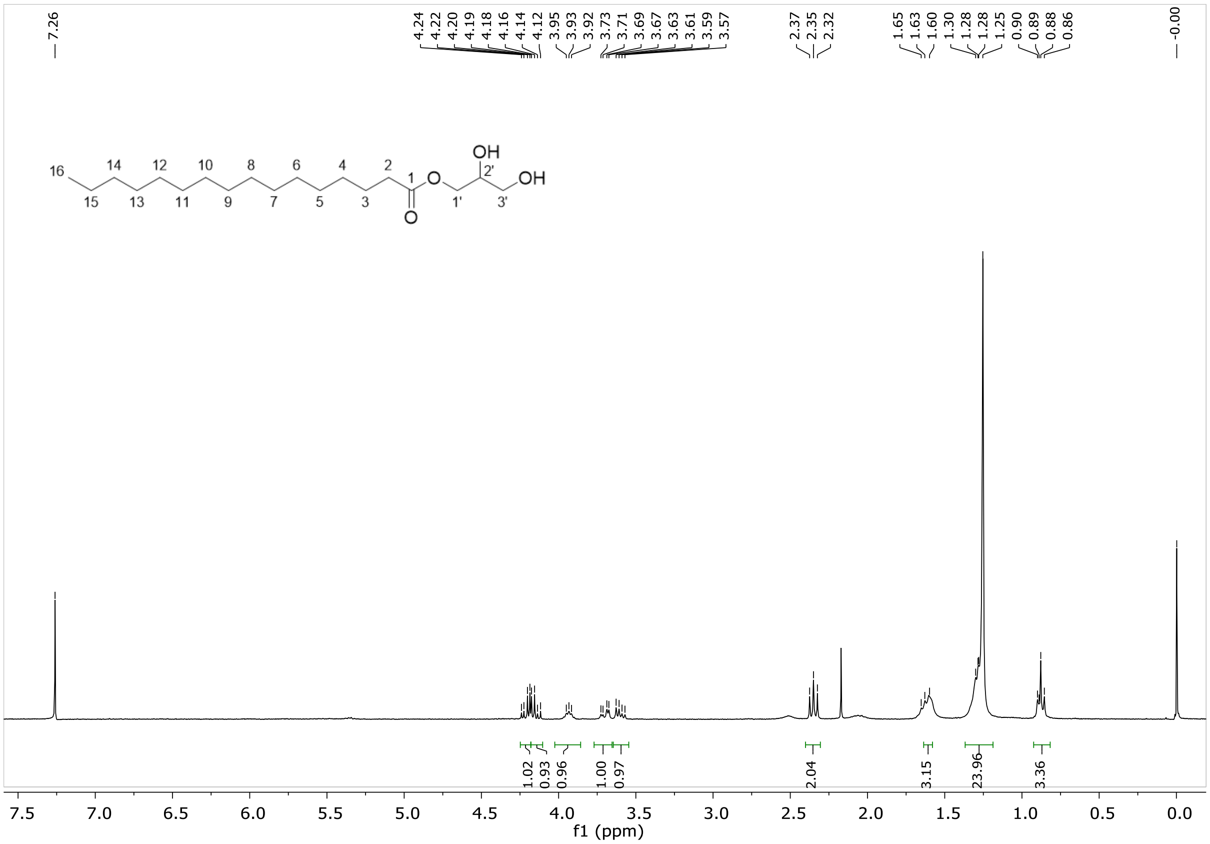

Figure S2.31: ^{1}H NMR spectrum (300 MHz, Chloroform-*d*) of compound 6 (Glyceryl palmitate)

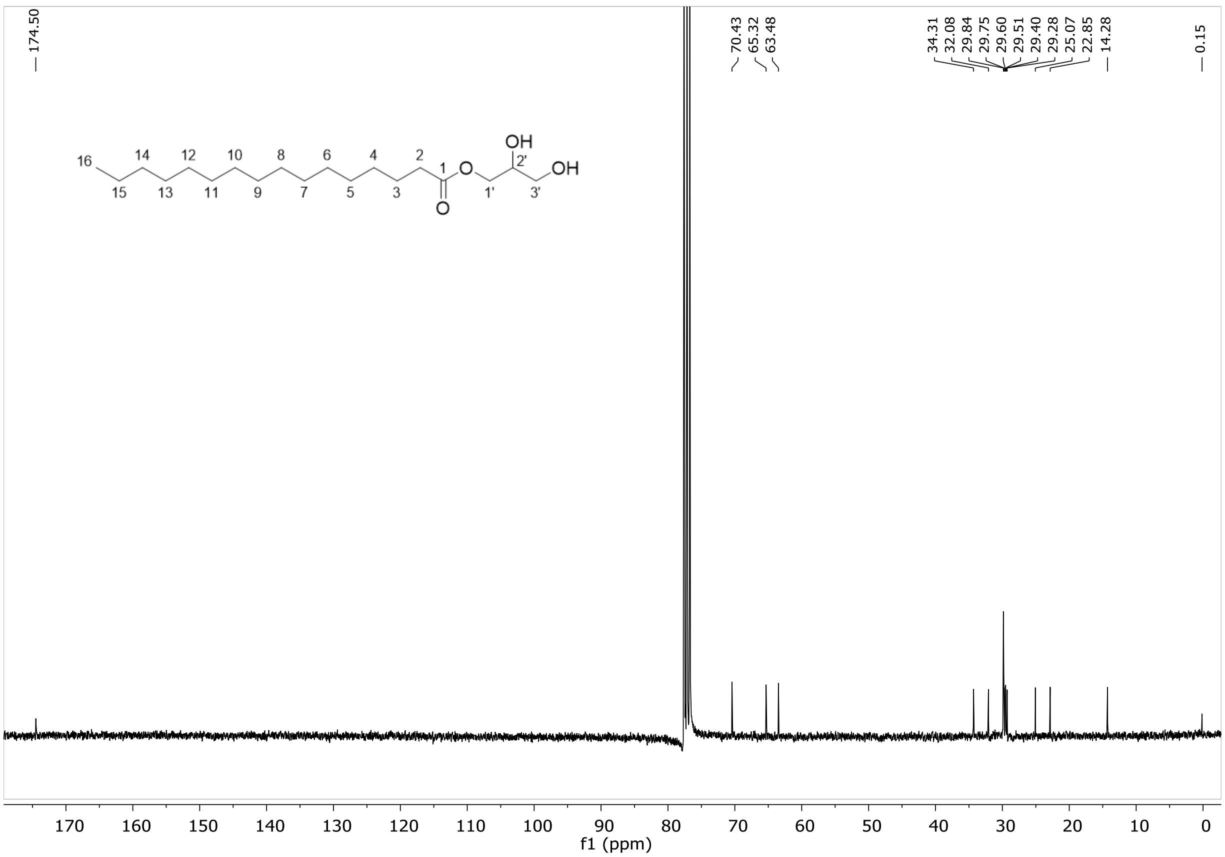

Figure S2.32: ^{13}C NMR spectrum (75 MHz, Chloroform-*d*) of compound 6 (Glyceryl palmitate)

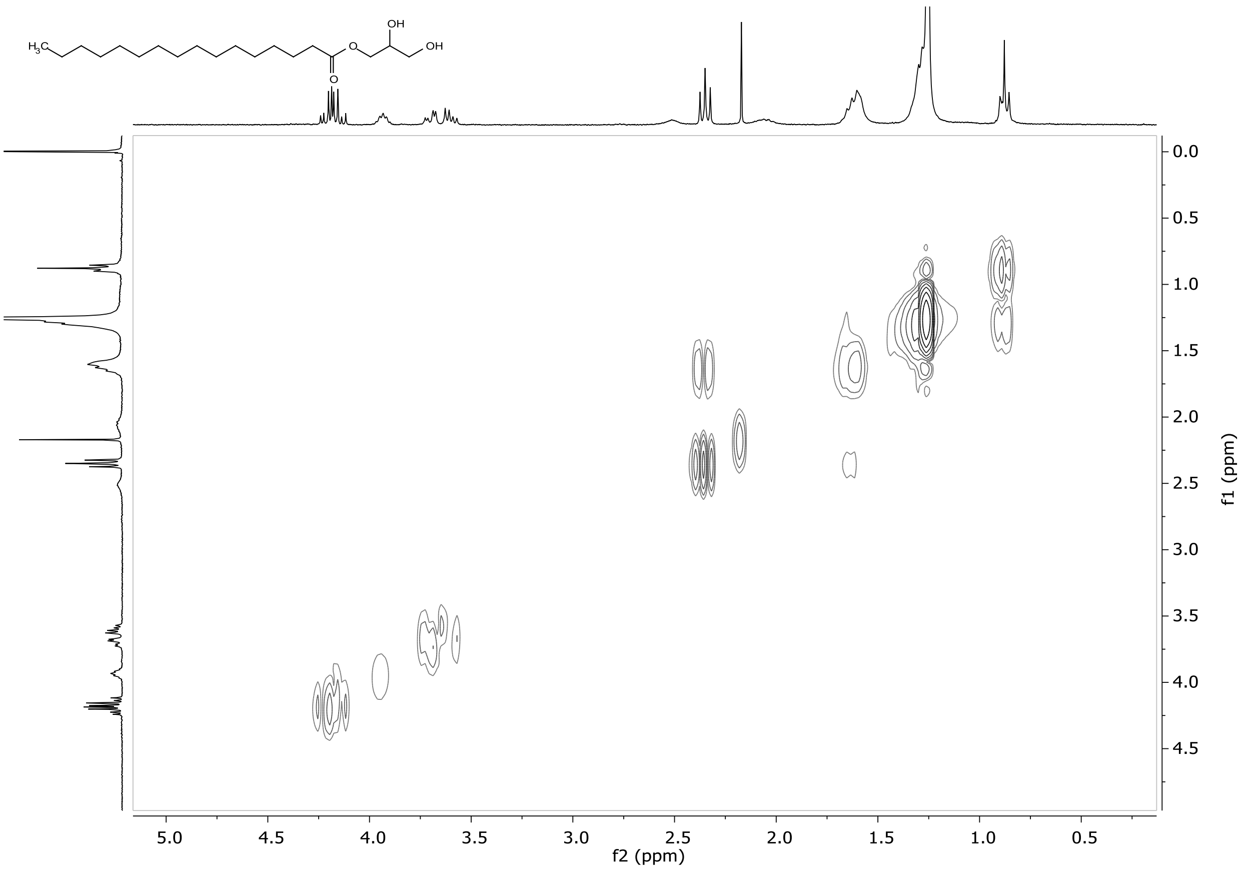

Figure S2.33: COSY spectrum of compound 6 (Glyceryl palmitate)

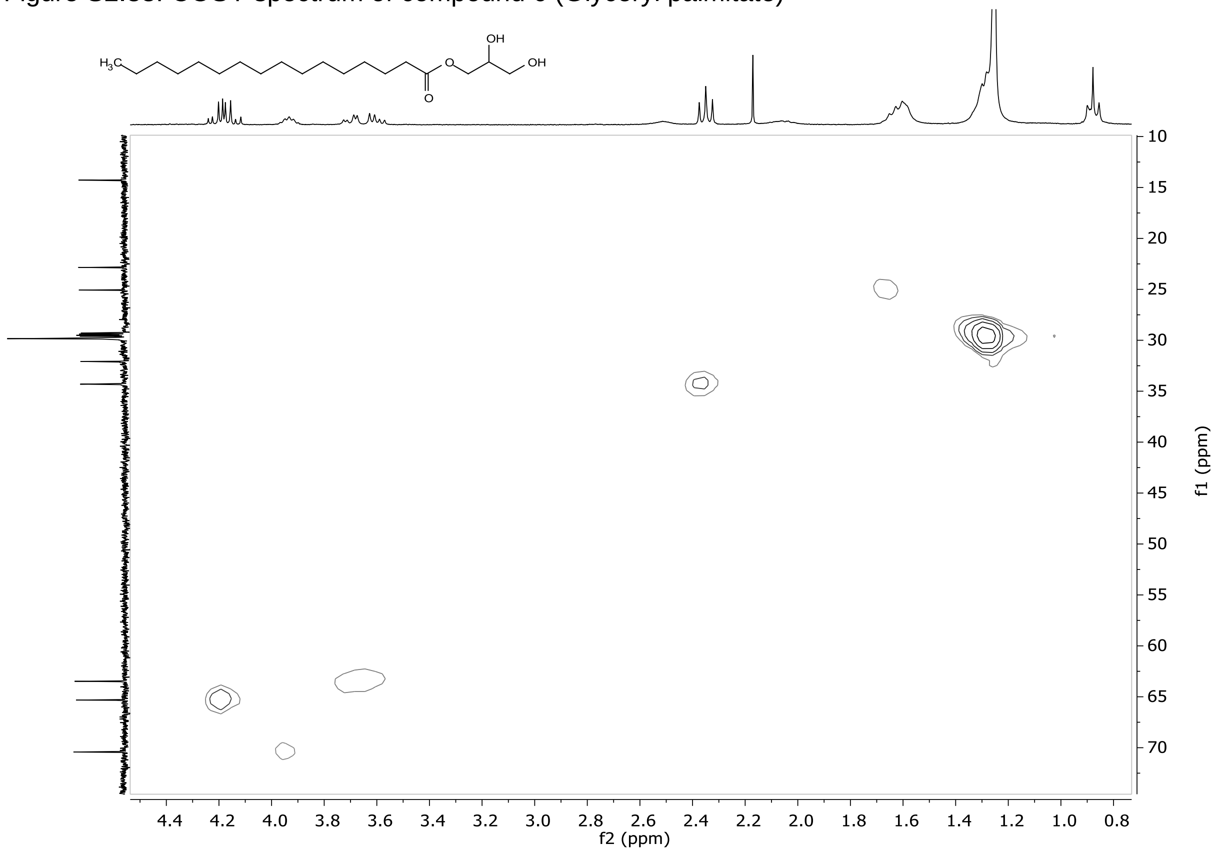

Figure S2.34: HSQC spectrum of compound 6 (Glyceryl palmitate)

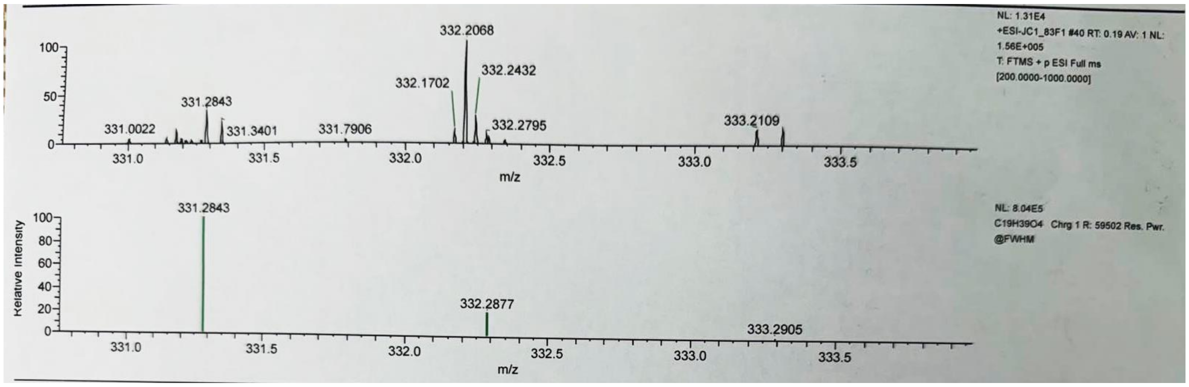

Figure S2.35: HRMS spectrum of compound 6 (Glyceryl palmitate)
