# Supplementary material for: Lutein derived from Xenostegia tridentata exhibits anticancer activities against A549 lung cancer cells via hyaluronidase inhibition
Source: PLoS One. 2024 Dec 16;19(12):e0315570. doi: 10.1371/journal.pone.0315570 (PMC11649105; doi:10.1371/journal.pone.0315570)
Supplement: S2 Fig — (PDF) [file pone.0315570.s003.pdf]

S3. HPLC chromatograms of the isolated compounds from ethyl acetate subfraction

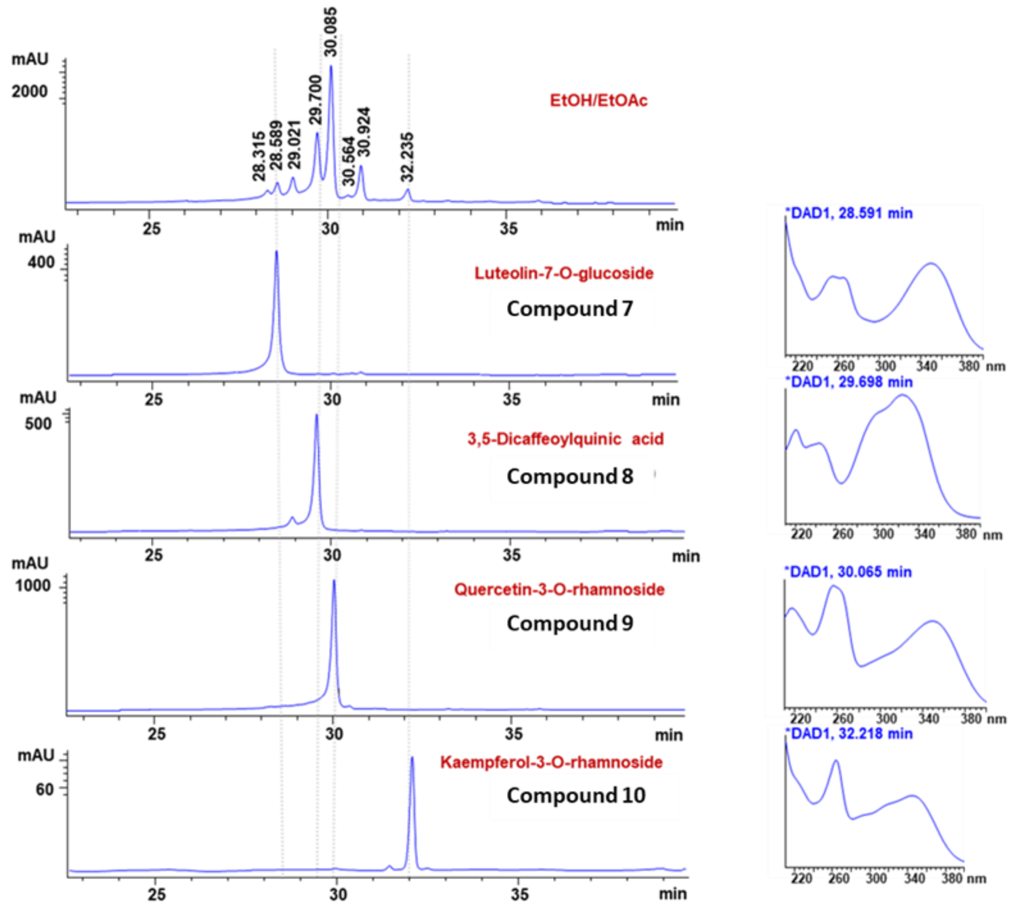

Figure S3: The HPLC analysis was performed by using a Kinetex C18 column (150 × 2.1 mm i.d., 2.6 µm). The separations were obtained by gradient elution using 0.1%v/v formic acid in milliQ water (solvent A) and acetonitrile (solvent B) as followed: 0 min, 95:5; 10 min, 95:5; 60 min, 30:70; 61 min, 1:99; 70 min, 1:99; 71 min, 95:5; 85 min, 95:5. The column temperature maintained at 40 °C. DAD spectra were recorded from λ = 200 to 400 nm and the chromatographic profiles were recorded at λ = 254 nm.
