# Supplementary material for: Lutein derived from Xenostegia tridentata exhibits anticancer activities against A549 lung cancer cells via hyaluronidase inhibition
Source: PLoS One. 2024 Dec 16;19(12):e0315570. doi: 10.1371/journal.pone.0315570 (PMC11649105; doi:10.1371/journal.pone.0315570)
Supplement: S3 Fig — (PDF) [file pone.0315570.s004.pdf]

S4. Docking simulation visualization

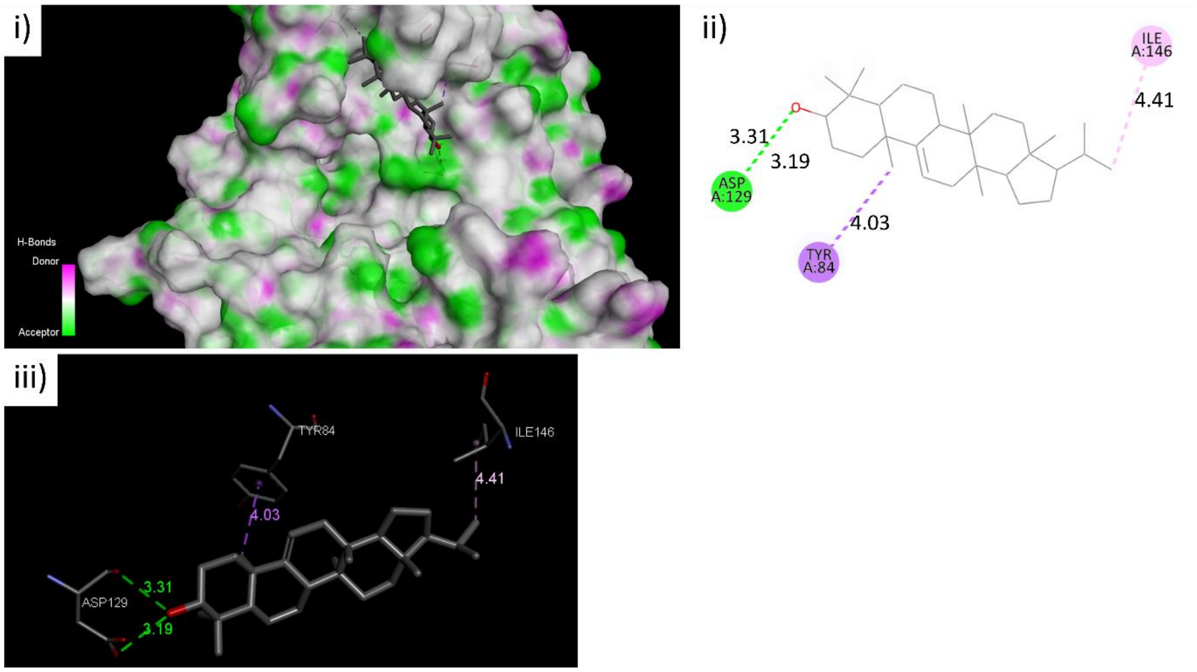

Figure S4A: i) Docking pose of fernenol (**1**) in Hyal-1 (PDB ID: 2PE4), ii) its 2D interaction diagram, and iii) its 3D representation of amino acid interactions**.** Note: light pink = π-alkyl/alky interaction, purple = π-σ interaction, and green = conventional hydrogen bond interaction.

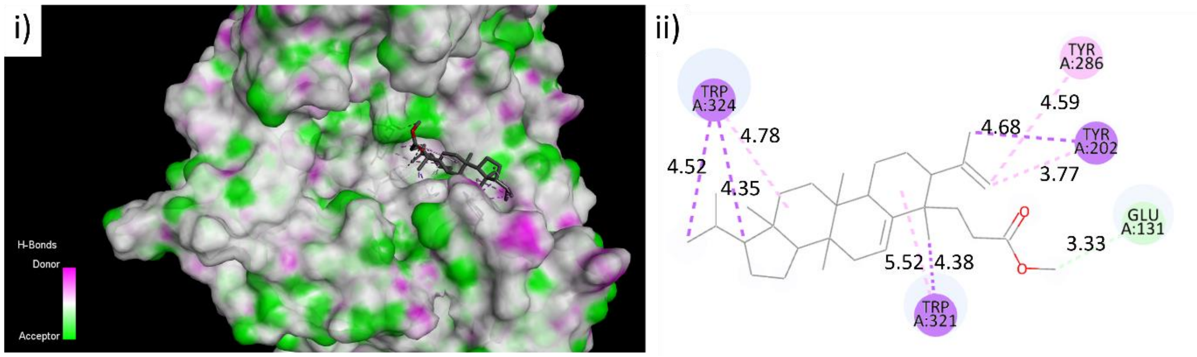

Figure S4B: i) Docking pose of methyl-3,4-seco-8*βH*-fernadienoate (**2**) in Hyal-1 (PDB ID: 2PE4) and ii) its 2D interaction diagram**.** Note: light pink = π-alkyl/alky interaction, purple = π-σ interaction, and light green = carbon hydrogen bond interaction.

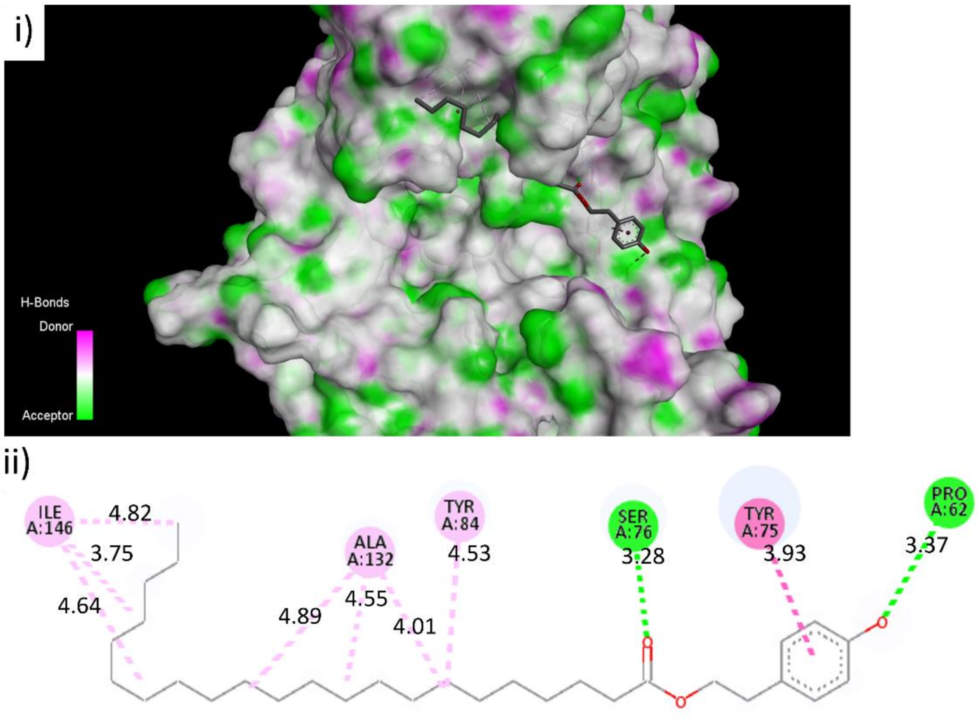

Figure S4C: i) Docking pose of 2(4'-Hydroxyphenyl)-ethyl behenate (**3**) in Hyal-1 (PDB ID: 2PE4) and ii) its 2D interaction diagram. Note: pink = π-π interaction, light pink = π-alkyl/alky interaction, and green = conventional hydrogen bond interaction.

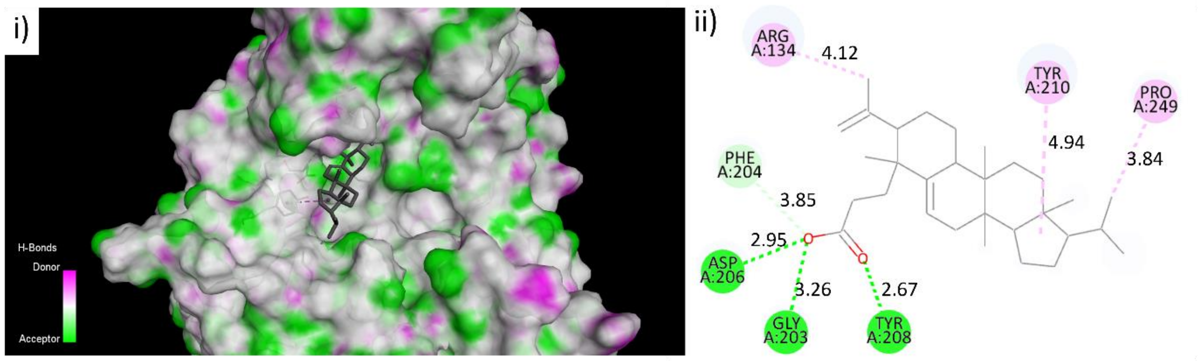

Figure S4D: i) Docking pose of 3,4-seco-8*βH*-fernadienoic acid (**4**) in Hyal-1 (PDB ID: 2PE4) and ii) its 2D interaction diagram**.** Note: light pink = π-alkyl/alky interaction, green = conventional hydrogen bond interaction, and light green = π-donor hydrogen bond interaction.

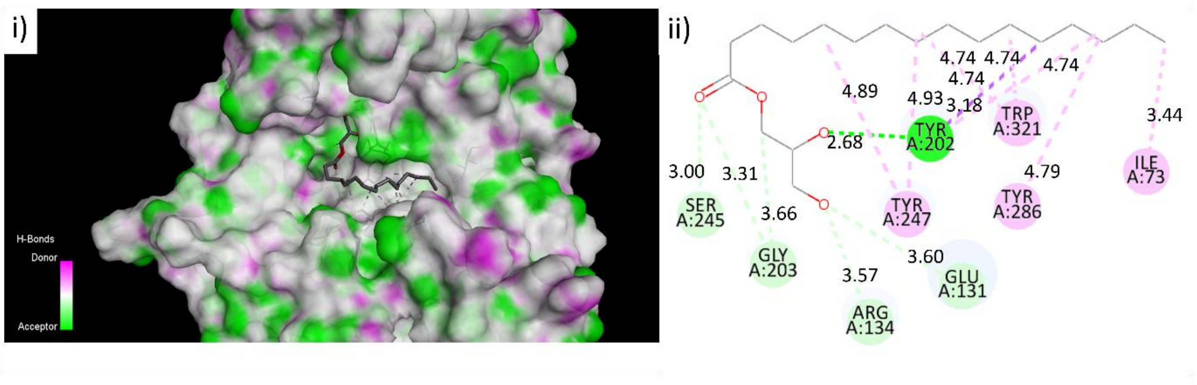

Figure S4E: i) Docking pose of glyceryl palmitate (**6**) in Hyal-1 (PDB ID: 2PE4) and ii) its 2D interaction diagram**.** Note: light pink = π-alkyl/alky interaction, purple = π-σ interaction, green = conventional hydrogen bond interaction, and light green = carbon hydrogen bond interaction.

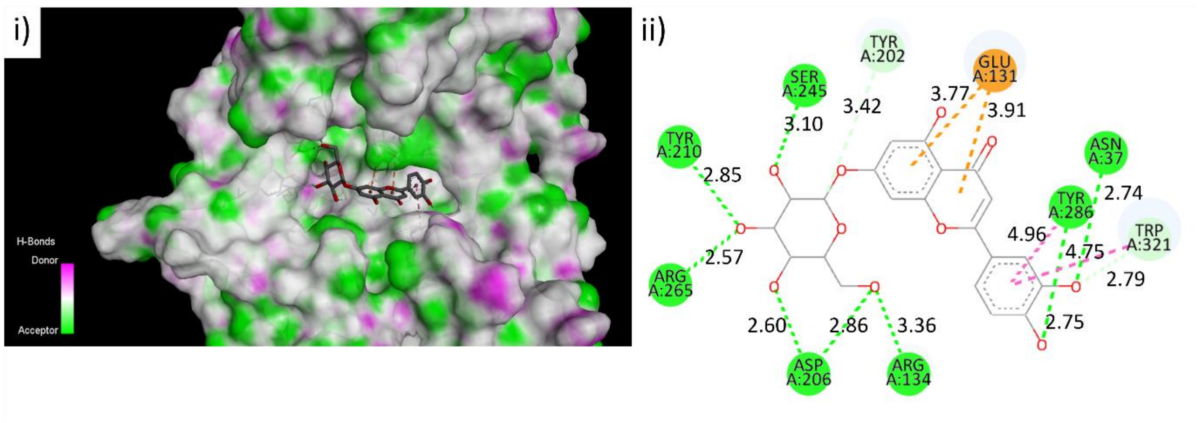

Figure S4F: i) Docking pose of luteolin-7-O-glucoside (**7**) in Hyal-1 (PDB ID: 2PE4) and ii) its 2D interaction diagram**.** Note: orange = π-anion interactions, pink = π-π interaction, green = conventional hydrogen bond interaction, and light green = carbon hydrogen bond interaction.

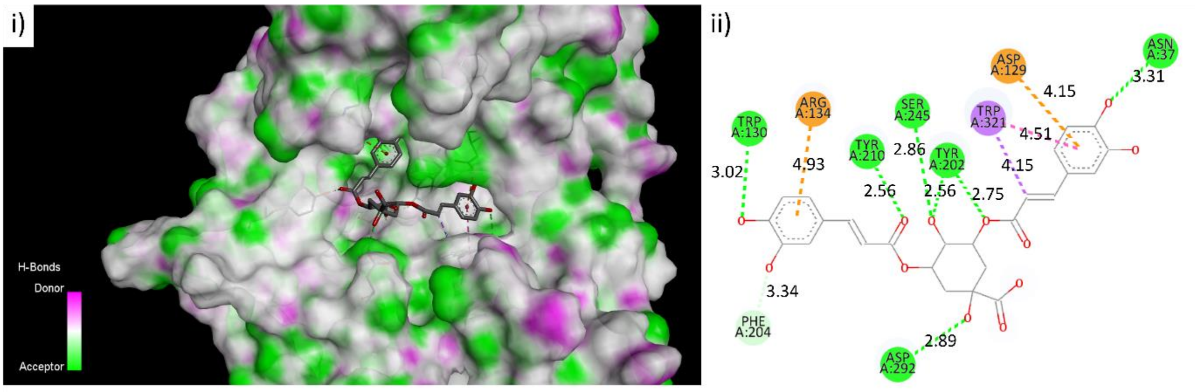

Figure S4G: i) Docking pose of 3,5-dicaffeoylquinic acid (**8**) in Hyal-1 (PDB ID: 2PE4) and ii) its 2D interaction diagram**.** Note: orange = π-anion/cation interactions, pink = π-π interaction, purple = π-σ interaction, green = conventional hydrogen bond interaction, and light green = π-donor hydrogen bond interaction.

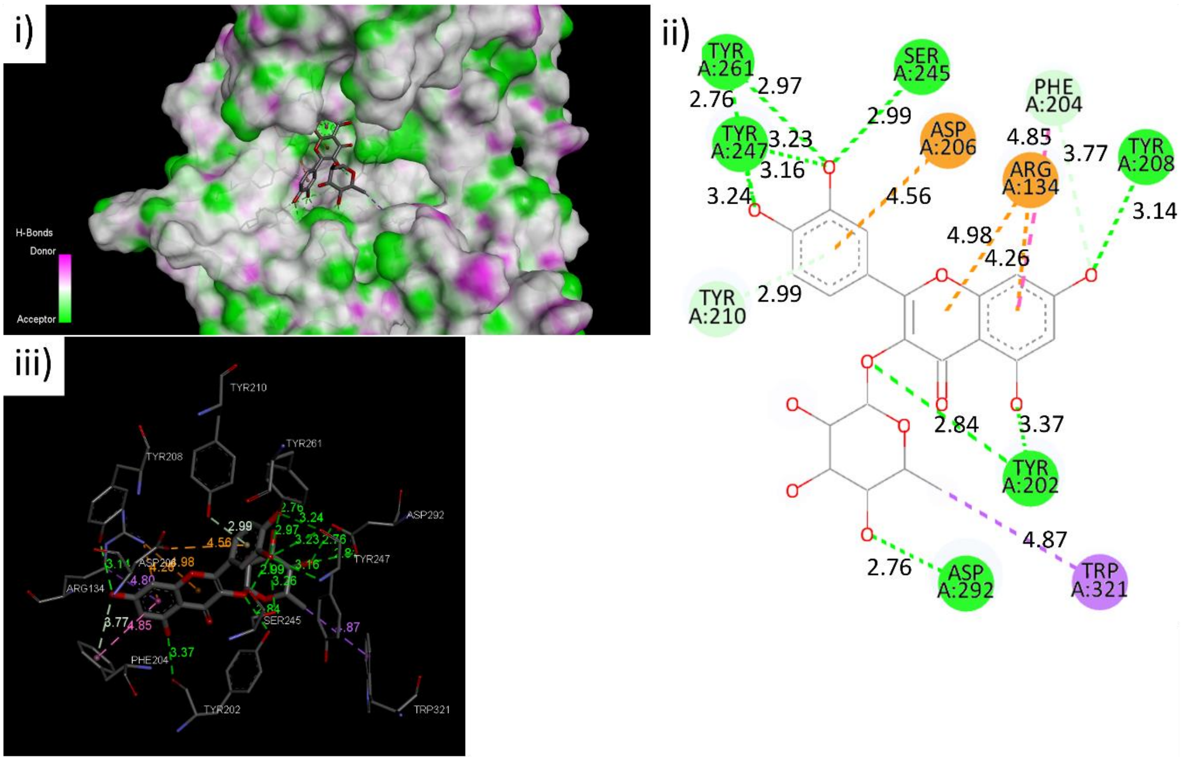

Figure S4H: i) Docking pose of quercetin-3-O-rhamnoside (**9**) in Hyal-1 (PDB ID: 2PE4), ii) its 2D interaction diagram, and iii) its 3D representation of amino acid interactions**.** Note: orange = π-anion/cation interactions, pink = π-π interaction, purple = π-σ interaction, green = conventional hydrogen bond interaction, and light green = π-donor hydrogen bond interaction.

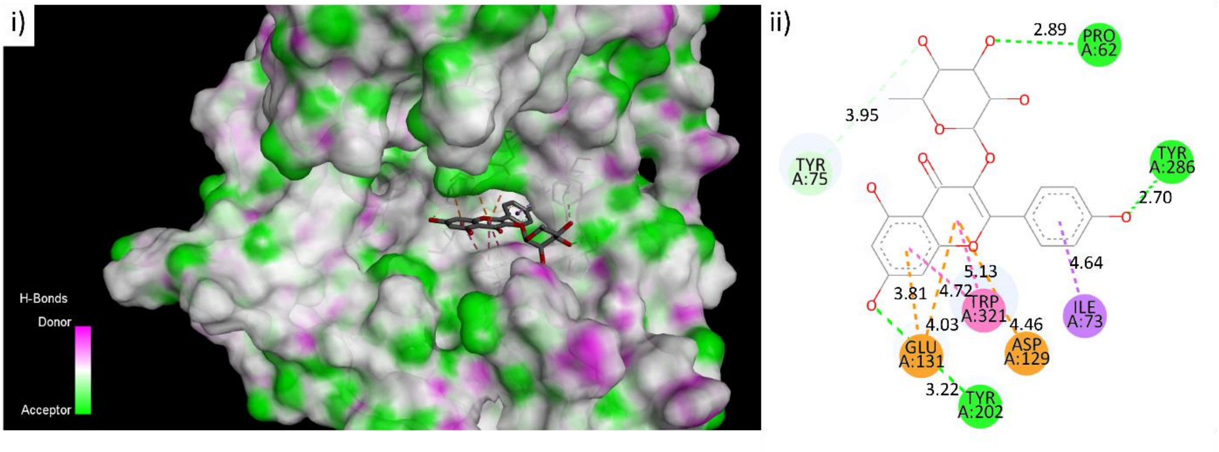

Figure S4I: i) Docking pose of kaempferol-3-O-rhamnoside (**10**) in Hyal-1 (PDB ID: 2PE4) and ii) its 2D interaction diagram**.** Note: orange = π-anion interactions, pink = π-π interaction, purple = π-σ interaction, and green = conventional hydrogen bond interaction.

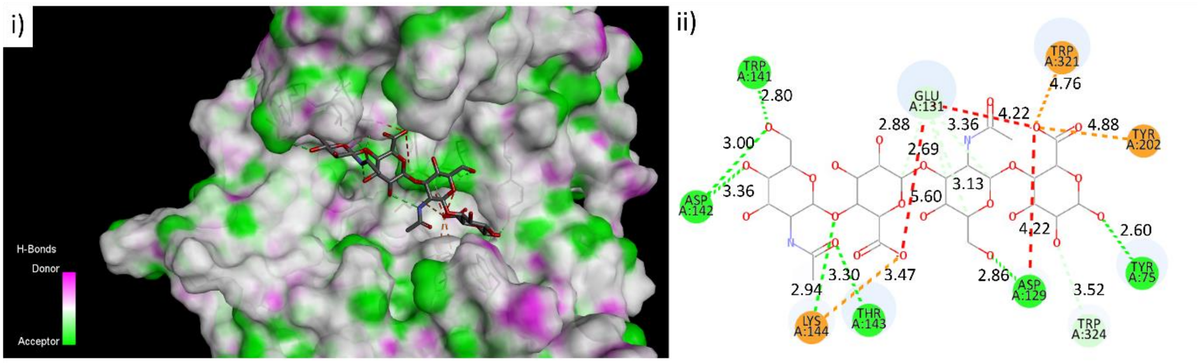

Figure S4J: i) Docking pose of hyaluronan in Hyal-1 (PDB ID: 2PE4) and ii) its 2D interaction diagram**.** Note: orange = π-anion interactions/attractive charge, green = conventional hydrogen bond interaction, light green = carbon hydrogen bond interaction, and red = unfavorable negative-negative electrostatic repulsion.
