# Supplementary material for: Lutein derived from Xenostegia tridentata exhibits anticancer activities against A549 lung cancer cells via hyaluronidase inhibition
Source: PLoS One. 2024 Dec 16;19(12):e0315570. doi: 10.1371/journal.pone.0315570 (PMC11649105; doi:10.1371/journal.pone.0315570)
Supplement: Attachment — Submitted filename: PLOS ONE.doc [file pone.0315570.s005.doc]

The study "Lutein from X. tridentat showing anti-cancer activity in lung cancer cells via hyaluronidase inhibition” is valuable and can be evaluated as potent to be published in PLOS ONE after major revisions.

Though the introduction is comprehensive, only the given isolated compounds are the only flavonoids present in the studied plant. Also, it unclear whether the plant is used in Indian medicine as a single plant or as a polyherbal mixture.

It is belter to use short sentences as it is too complicated to read at times. For example, page 5: lines 5-9 is one sentence.

The isolation method should be clarified further. It is also unclear why the authors used ethanol to for the extraction procedure. What was the final remaining extraction upon fractionation. The fractionation procedure should be clearly explained.

**Other comments**

Abstract

The results should indicate anticancer activity. Give future direction using one line.

Introduction:
The authors should clarify is this the only mechanism by which NSCLC is upregulated. The authors should highlight the main components of the signaling pathway. It is unclear that the authors who studied, R. madagascariensis, managed to isolate a compound important for the inhibition.

Material & Method

Give the coordination of the collected area.

Give the temperature of the evaporator.

MTT assay – after how many hours of culture, the authors conducted the MTT assay.

It authors could have conducted a siRNA inhibitory transfection to confirm the

Results of LWW hyaluronan.

What was the column size used for isolation procedure.

Give details of using this particular protein for molecular docking (see Integration of in vitro and in-silico analysis of *Caulerpa racemosa* against antioxidant, antidiabetic, and anticancer activities. 2022 *Sci Rep* 12, 20848 (2022). <https://doi.org/10.1038/s41598-022-24021-y>

The statistical analysis should be more descriptive.

Results and Discussion:

The authors should have used a standard drug to compare anticancer properties. Further, it is better to confirm antiproliferative assay using another technique.

Data visualization could be improved.

Fig 2. Photomicrographs are unclear. Introduce photos and give magnification.

Obtained data should be discussed.

According to the results, all the compounds have been isolated previously from other plants. Are there any new compounds isolated from the studied plant.

Give the recorded therapeutic activities of the compound. When discussing, the interrelation the results are important.

. The docking analysis should discuss about van der Val bonding patterns and number of hydrogen bonds formed. See “*In silico* study of SARS-CoV-2 Spike protein RBD and human ACE-2 affinity dynamics across variants and Omicron sub-variants. Journal of Medical Virolog (2022). y. 94 (1), e28406 <https://doi.org/10.1002/jmv.28406>.

Also, Since the title is Docking Analysis, a much more detailed discussion is required in this section.

Figure 9 – figure ledged should explain all the figures representing the figure.
